# Ecological niche modeling for conservation planning of an endemic snail in the verge of becoming a pest in cardamom plantations in the Western Ghats biodiversity hotspot

**DOI:** 10.1002/ece3.2368

**Published:** 2016-08-18

**Authors:** Sandeep Sen, Kadukothanahally Nagaraju Shivaprakash, Neelavara A. Aravind, Gudasalamani Ravikanth, Selvadurai Dayanandan

**Affiliations:** ^1^ Suri Sehgal Centre for Biodiversity and ConservationAshoka Trust for Research in Ecology and the EnvironmentRoyal EnclaveSrirampuraBangaloreKarnataka560064India; ^2^ Manipal UniversityManipalKarnataka576504India; ^3^ Department of BiologyConcordia UniversitySherbrooke Street WestMontrealQuebecH4B 1R6Canada; ^4^ Quebec Centre for Biodiversity ScienceMontrealQuebecH3A 1B1Canada; ^5^ School of Ecology and ConservationUniversity of Agricultural SciencesHebbalBangaloreKarnataka560063India

**Keywords:** Biological invasion, *Elettaria cardamomum*, endemic snail, *Indrella ampulla*, MIGCLIM, niche overlap, Western Ghats

## Abstract

Conservation managers and policy makers are often confronted with a challenging dilemma of devising suitable strategies to maintain agricultural productivity while conserving endemic species that at the early stages of becoming pests of agricultural crops. Identification of environmental factors conducive to species range expansion for forecasting species distribution patterns will play a central role in devising management strategies to minimize the conflict between the agricultural productivity and biodiversity conservation. Here, we present results of a study that predicts the distribution of *Indrella ampulla*, a snail endemic to the Western Ghats biodiversity hotspot, which is becoming a pest in cardamom (*Ellettaria cardamomum*) plantations. We determined the distribution patterns and niche overlap between *I. ampulla* and *Ellettaria cardamomum* using maximum entropy (MaxEnt) niche modeling techniques under current and future (2020–2080) climatic scenarios. The results showed that climatic (precipitation of coldest quarter and isothermality) and soil (cation exchange capacity of soil [CEC]) parameters are major factors that determine the distribution of *I. ampulla* in Western Ghats. The model predicted cardamom cultivation areas in southern Western Ghats are highly sensitive to invasion of *I. ampulla* under both present and future climatic conditions. While the land area in the central Western Ghats is predicted to become unsuitable for *I. ampulla* and *Ellettaria cardamomum* in future, we found 71% of the Western Ghats land area is suitable for *Ellettaria cardamomum* cultivation and 45% suitable for *I. ampulla*, with an overlap of 35% between two species. The resulting distribution maps are invaluable for policy makers and conservation managers to design and implement management strategies minimizing the conflicts to sustain agricultural productivity while maintaining biodiversity in the region.

## Introduction

Endemic species that are at the early stages of becoming pests of agricultural crops pose a tremendous challenge to conservation managers and policy makers in devising suitable strategies to address conflicting demands between sustenance of agricultural productivity and biodiversity conservation. Implementing eradication measures may lead to extinction of endemic species. Alternatively, if left uncontrolled, these species may become pests with significant negative impact on the economy and livelihood of farming communities. Thus, implementing preventive measures at early stages remains as the best management strategy to avoid conflicts between the maintenance of livelihood of farming communities and biodiversity conservation (Thuiller et al. 2005). Recent advances in ecological niche modeling techniques provide an unprecedented opportunity to predict geographic distribution patterns of species and determine habitats vulnerable to the spread of pest species (Peterson and Vieglais 2001; Ganeshaiah et al. 2003; Peterson 2003; Thuiller et al. 2005; López‐Darias et al. 2008; Trethowan et al. 2010).

The predictions based upon ecological niche models have been successfully used to integrate physiological threshold of species, land cover, and remote sensing data to model and then predict sites that are highly sensitive to invasion of pests (Byers et al. 2002; Guisan and Thuiller 2005; Aragón et al. 2010; Trethowan et al. 2010). In addition, the ecological niche models are also useful for predicting areas that are ecologically suitable for the establishment of invasive and pest species under the future climatic scenarios (Ficetola et al. 2007; Larson and Olden 2012; O'Donnell et al. 2012). However, the projected distribution maps of invasive and pest species produced through ecological niche modeling methods alone may not be realistic without taking the dispersal ability of a species to colonize available habitats in future. In general, ecological niche models predict potential distribution of species assuming unrestricted migration scenario, which is unrealistic for those species with the limited dispersal capacity. Thus, it is crucial to integrate dispersal constrains of target species to realistically predict the future distribution pattern under the anticipated climate change scenarios. Recently, several tools have been developed to take the dispersal constrains of species into account, and hence, one can predict more realistic future distribution of invasive and native pest species under the expected climate change scenarios (Engler et al. 2012; Bateman et al. 2013).

**Figure 1:**
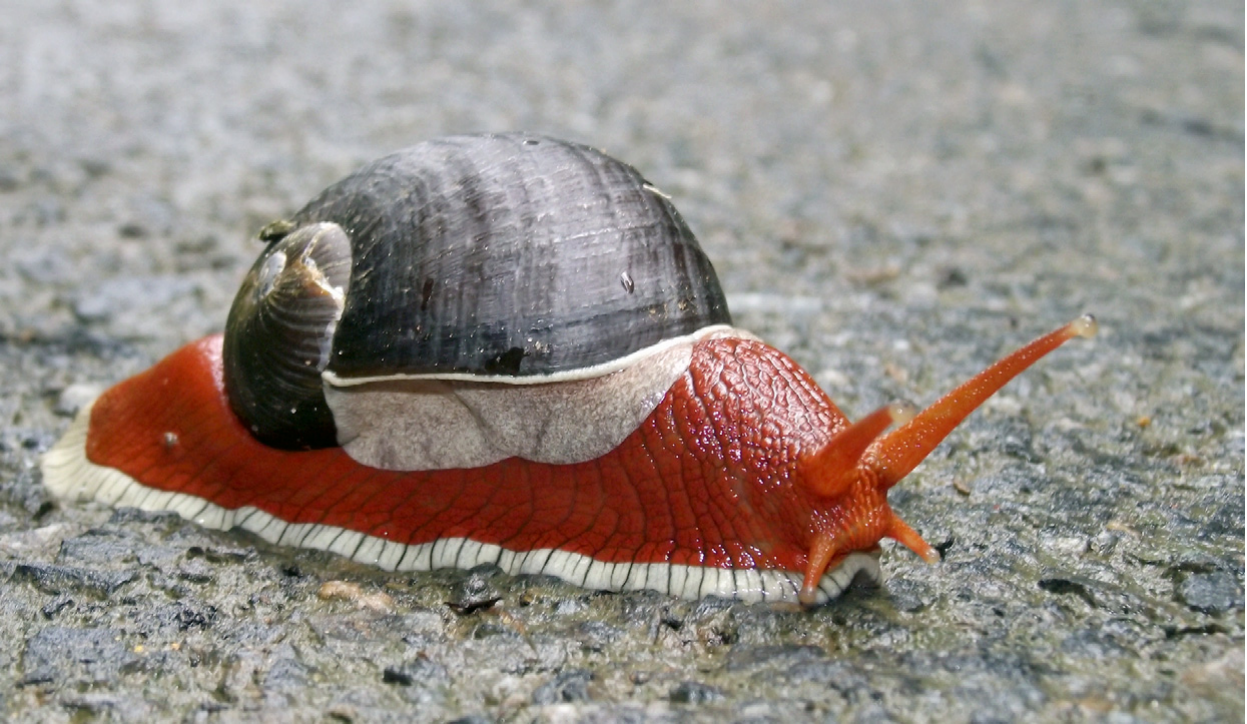
Image of endemic land snail *Indrella ampulla* (copyright Anoop. N.R).

*Indrella ampulla* Benson (Gastropoda: *Ariophantidae*), a large monotypic species of land snail endemic to the Western Ghats, India (Mavinkuruve et al. 2004; Aravind et al. 2009) (Fig. 1 and Fig. S1A in Appendix S1), is generally found in bioclimatically stable, wet woodland habitats such as tropical rain forests in the region (Aravind et al. 2009). *Indrella ampulla* also serves as an important food source of an endangered cane turtle (*Vijayachelys sylvatica*) (Deepak 2009). During the last few decades, large areas of tropical rain forests in the Western Ghats have been converted to cardamom (*Elettaria cardamomum*) plantations. *Indrella ampulla* has become a nocturnal opportunistic feeder of flowers and fruits of cardamom plants, and thus becoming a pest causing significant impact on the productivity of cardamom plantations (Sudhi 2010).

Since cardamom is one of the economically important major plantation crops extensively grown in southern and central region of the Western Ghats, there is a concern that *I. ampulla* may become a serious pest spreading into other cardamom growing areas leading to serious economic losses. Consequently, measures taken by farmers to control snails in cardamom plantations may negatively affect the natural populations of *I. ampulla*. Thus, predicting the factors that influence the distribution of *I. ampulla* in Western Ghats and surrounding cardamom cultivation areas that may become susceptible to invasion by *I. ampulla* is of particular interest for developing management strategies to avoid conflict between cardamom growers and biodiversity conservation.

The objectives of this study were to (1) build predictive models of *I. ampulla* distribution in Western Ghats and identify environmental variables that best predict its distribution using maximum entropy (MaxEnt) modeling approach, (2) predict the suitable areas for cardamom (*E. cardamomum*) cultivation in Western Ghats, (3) assess the extent of overlap between highly suitable habitat of *I. ampulla* and cardamom cultivation areas in Western Ghats under the present and future climatic scenarios, (4) test whether establishment success and potential invasiveness of *I. ampulla* associated with predicted habitat suitability by combining habitat suitability prediction and demographic parameters, and (5) identify cardamom cultivation areas highly vulnerable to *I. ampulla* invasion by developing an invasion risk map of *I. ampulla* to cardamom cultivation areas under the present and future climatic scenarios in Western Ghats.

## Materials and Methods

### Occurrence records and predictor variables

The occurrence records of *I. ampulla* and *E. cardamomum* were gathered from both field survey (2009–2011) and secondary data including the published literature (Kumar et al.^1995^; Prasath and Venugopal 2004; Kuriakose et al. 2009) (Appendix S1 for details). We used 32 environmental variables to predict the potential distribution of suitable habitats for *I. ampulla* and *E. cardamomum* in Western Ghats under the present and future (2020–2080) climatic scenarios. Of these variables, 19 were bioclimatic (Hijmans et al. 2005), seven were edaphic (FAO 2012), three were related to global aridity and evapotranspiration (Zomer et al. 2007, 2008), and three were topographic layers (Appendix S1, Table S1). These layers had resolution of 30 arc seconds (1000 m). All layers were clipped to Indian subcontinent and projected to geographic coordinate system WGS84.

Since multicollinearity between explanatory variables can lead to inaccurate prediction by excluding significant predictive variables (Graham 2003), pairwise Pearson correlation coefficients were calculated for all combinations of variables, and one variable from any pair of variables with a Pearson correlation coefficient greater than 0.7 (*r* ^2^ > 0.49) was removed from consideration (McDowell et al. 2014) (Appendix S1, Table S1). The variables chosen to remove were based on their biological relevance to the species. For example, pH was highly correlated with total exchangeable bases (*r* = 0.893); from this pair, total exchangeable bases were removed, because pH in general is known to affect the shell formation in many mollusks (Hunter 1990; Økland 1992; Glass and Darby 2009; Beniash et al. 2010). Finally, we retained 21 variables to model suitable habitats for *I. ampulla* and *E. cardamomum* (Appendix S1, Tables S1 and S2).

### Modeling habitat suitability

We used maximum entropy algorithm as implemented in MaxEnt software (version 3.3.3k; Phillips et al. 2006) to prepare maps of habitat suitability for *I. ampulla* and *E. cardamomum* using occurrence records and environmental layers (See Appendix S1 for further details on modeling method).

Based on the predicted maps, we selected eight sites belonging to different habitat suitability categories (highly suitable, medium and poor, or unsuitable) to collect demographic data of *I. ampulla* (Appendix S5, Table S5) and evaluated the predictions whether *I. ampulla* populations located in highly suitable habitat had higher number of adults and higher reproductive rates (juveniles/adult) as compared to populations located in poor or unsuitable habitats (See Appendix S5 for a full description of the methodology used).

### Overlap of suitable habitats

The overlap between suitable habitats of *I. ampulla* and *E. cardamomum* cultivation areas under the present climatic scenario was assessed by comparing the present habitat suitability maps. To do this, we converted best performing habitat suitability model of *E. cardamomum* and *I. ampulla* to a presence–absence maps using the maximum sensitivity plus specificity threshold (Table 1). Then, we superimposed the converted presence–absence habitat suitability map of *I. ampulla* with habitat suitability map of *E. cardamomum* to determine areas of overlap. We calculated the number of pixels in suitability map of *I. ampulla* overlapped with suitability maps of *E. cardamomum* cultivation areas across latitude in Western Ghats. We also calculated the proportional overlap (Reyers et al. 2000) by taking the number of pixels that overlapped divided by the maximum number of overlapping pixels possible (i.e., the map with the smallest number of pixels). (Appendix S6 for a full description of the methodology used to predict the habitat overlap for *I. ampulla* and *E. cardamomum* in future climate scenario).

**Table 1:** The mean area under curve (AUC) and true skill statistics (TSS) values from 50 replicate models of *Indrella ampulla* and *Ellettaria cardamomum*

Species	Time period	AUC_TRAIN_	AUC_TEST_	TSS	MTSS threshold used for categorical classification
*Indrella ampulla*	Current	0.994 (0.000)	0.991 (0.008)	0.976 (0.017)	0.339
	Future	0.994 (0.000)	0.990 (0.007)	0.973 (0.021)	0.417
*Ellettaria cardamomum*	Current	0.991 (0.000)	0.986 (0.013)	0.962 (0.010)	0.183
	Future	0.991 (0.000)	0.986 (0.014)	0.961 (0.012)	0.078

Values in parenthesis represent standard deviation. MTSS, maximum training sensitivity plus specificity.

### Prediction of invasion risk under the present climate scenario

We developed a model for assessing *I. ampulla* invasion risk to areas where *E. cardamomum* is cultivated. The model consisted of three components: (1) climate suitability of *E. cardamomum* cultivation areas to *I. ampulla*, (2) land cover transformation, and (3) distance of pixel predicted to be suitable for *E. cardamomum* cultivation to *I. ampulla* occurrence record. We superimposed categorical suitability maps of *I. ampulla* with the best‐performed presence–absence suitability maps of *E. cardamomum* to produce the map showing climatic suitability of *E. cardamomum* cultivation areas to *I. ampulla* distribution. Since *I. ampulla* is native to tropical rain forest of Western Ghats, we assigned tropical broad‐leaved forest in Western Ghats to values of 1 in MODIS land cover map of 2011 obtained from NEO NASA earth observations (http://neo.sci.gsfc.nasa.gov). The land transformed to other uses and degraded land classes were set to a value of 0. This resulted in land cover transformation map. We calculated the Euclidian distance between the nearest *I. ampulla* occurrence record (using ArcGIS Version10.0 (ESRI. 2012) Geospatial Modelling Environment) and for each pixel in a habitat suitability map of *E. cardamomum* cultivation areas in Western Ghats. We then rescaled the Euclidian distance between 0 and 1 so that furthest distance of *E. cardamomum* pixel to *I. ampulla* occurrence in map had a value of 0 and closest had a value of 1. This map is referred to as distance map. Finally, we produced an invasion risk probability map by adding the climatic suitability map of *E. cardamomum* cultivation areas to *I. ampulla*, the land cover transformation map, and the distance map and then rescaled the values between 0 and 1. The probability of invasion risk ranged between 0 (low risk) and 1 (high risk). All calculations and mapping were performed in ArcGIS version 10.0 (ESRI 2012). Finally, we calculated the number of pixels predicted to experience high and low risk of invasion along the latitude (south to north) in Western Ghats.

### Incorporating dispersal constrains and predicting invasion risk under future climatic scenario

We used a cellular automation model as implemented in the MIGCLIM R package (Engler and Guisan 2009; Engler et al. 2009) to incorporate dispersal constrains of *I. ampulla* and predict potential distribution and invasion under future climatic scenarios for the period 2020–2080. MIGCLIM was initialized to model the dispersal of *I. ampulla* over a period of 80 years (2000–2080) under three dispersal scenarios: (1) unlimited dispersal (species can disperse to any suitable cell); (2) no dispersal, and (3) limited dispersal with strong barriers (species can disperse following the MIGCLIM simulation, but are affected by dispersal barriers such as land cover classes). The MIGCLIM models were parameterized by following settings: rcThreshold = 600, encChgSteps = 4, dispSteps = 20, iniMatAge = 1, propagate production = 1, lddFreq = 0.05, lddMinDist = 2, lddMaxDist = 0, replicates = 5 (Engler et al. 2009). Since *I. ampulla* is native to tropical rain forest of Western Ghats, we assigned tropical broad‐leaved forest in Western Ghats to values of 0 in MODIS land cover map of 2011 obtained from NEO NASA earth observations (http://neo.sci.gsfc.nasa.gov). All other transformed and degraded land classes to value of 1. This transformed land cover map was used as barrier layer in MIGCLIM analysis considering cells having value of 1 as strong barriers.

Finally, we produced an invasion risk probability map of *I. ampulla* to *E. cardamomum* cultivation areas in Western Ghats under future climatic scenario by superimposing dispersal‐constrained future suitability maps (2020–2080) of *I. ampulla* on the future suitability maps (2020–2080) of *E. cardamomum* cultivation areas in Western Ghats (Appendix S1 for a full description of the methodology used to generate suitability maps under future climate scenario (2020–2080) for *I. ampulla* and *E. cardamomum*). All calculations and mapping were performed in ArcGIS version 10.0.

## Results

### Model calibration and prediction of most relevant environmental variables

The model performance was found excellent for *I. ampulla* in both climatic scenarios (current and future [2020–2080]). The average values of AUC and TSS obtained from 50 replicated models (evaluated using 30% of records) were very high (current: AUC_TRAIN_ = 0.994 ± 0.000, AUC_TEST_ = 0.991 ± 0.008; future: AUC_TRAIN_ = 0.994 ± 0.000, AUC_TEST_ = 0.990 ± 0.007) and (TSS: current = 0.976 ± 0.017; future = 0.973 ± 0.021) (Table 1) (see Appendix S4, Table S4 for model calibration and performance results of *E. cardamomum*). Among the 18 input environmental variables, five variables were significant contributors to the distribution model of *I. ampulla*: Precipitation of warmest quarter (62.52%) and isothermality (11.54) had highest contribution to the model followed by topsoil cation exchange capacity (CEC) (7.88), elevation (5.56%), and global aridity index (5.01%). All five variables together explained 92.51% of variation in the model (Table 2). The remaining 13 variables collectively contributed to 8.5% of variation in the habitat suitability model. Considering the permutation importance, same five variables had maximum influence on habitat suitability model (Table 2). Even in future climatic scenario, same variables except global aridity (which was replaced by maximum temperature of warmest month) had highest percentage contribution to the model (Table 2). These five variables were also associated with an increased likelihood of the environment being suitable for *I. ampulla* distribution in Western Ghats as indicated by their response curves (Appendix S3, Fig. S4A–E). Jackknife test for environmental variables showed high values of training and test gain and high‐test AUC values for the same five environmental variables (Appendix S3, Fig. S4F–H). See Appendix S3, Table S3 and Fig. S3A–H for results of important predictors of habitat suitability for *E. cardamomum*.

**Table 2:** Variables with the percent contribution and permutation importance in the predicted distribution of species

Variables	Current				Future			
	*Indrella ampulla*		*Ellettaria cardamomum*		*Indrella ampulla*		*Ellettaria cardamomum*	
	% contribution	Permutation	% contribution	Permutation	% contribution	Permutation	% contribution	Permutation
Bioclim2	1.57	3.22	0.82	0.83	1.48	5.99	4.33	0.08
Bioclim3	**11.54**	**16.33**	**46.90**	**54.13**	**10.43**	**4.74**	**40.49**	**49.25**
Bioclim5	**3.26**	**4.95**	**15.22**	**6.03**	**4.16**	**5.61**	**13.41**	**15.79**
Bioclim6	0.00	0.01	0.01	0.14	0.00	0.00	0.07	0.32
Bioclim15	0.01	0.00	0.45	0.55	0.23	1.76	**5.40**	1.15
Bioclim16	0.38	0.00	0.00	0.00	2.03	0.27	0.81	0.08
Bioclim17	0.02	0.23	0.08	0.16	0.14	4.09	0.04	0.24
Bioclim18	0.13	1.73	0.58	7.41	0.25	1.87	2.13	**22.82**
Boiclim19	**62.52**	**6.07**	**17.41**	**3.38**	**63.30**	**3.19**	**18.82**	0.12
AET	–	–	0.11	0.19	–	–	–	–
PET	–	–	0.05	0.00	–	–	–	–
AI	**5.01**	**0.39**	**6.53**	**4.06**	–	–	–	–
T_OC	0.17	3.26	0.55	0.94	0.00	0.00	1.56	0.24
T_CaCO3	0.73	0.32	1.70	**4.40**	0.40	1.75	3.01	0.20
T_CEC	**7.88**	**54.46**	1.29	**14.80**	**11.85**	**66.15**	0.48	**4.32**
T_PH_H2O	0.09	2.07	0.20	0.00	2.12	0.58	0.08	0.01
T_ECE	0.00	0.00	1.86	0.00	0.12	0.09	0.48	4.35
T_BD	–	–	0.11	0.00	–	–	0.05	0.21
DEM	**5.56**	**5.44**	**5.07**	1.91	**2.80**	**3.39**	**5.81**	**4.67**
Aspect	0.39	1.11	0.00	0.00	0.57	0.48	0.29	0.05
Slope	0.73	2.49	1.05	1.10	0.11	0.05	2.12	0.37

Variables with more than 2.5% contribution and permutation are given in bold, “–” indicates variables not used in model prediction. For variables abbreviations see Table S2. Note. some variables are more important in current and some are gaining its importance while projecting, because few variables were omitted during future projection analysis.

### Distribution of suitable habitats

**Figure 2:**
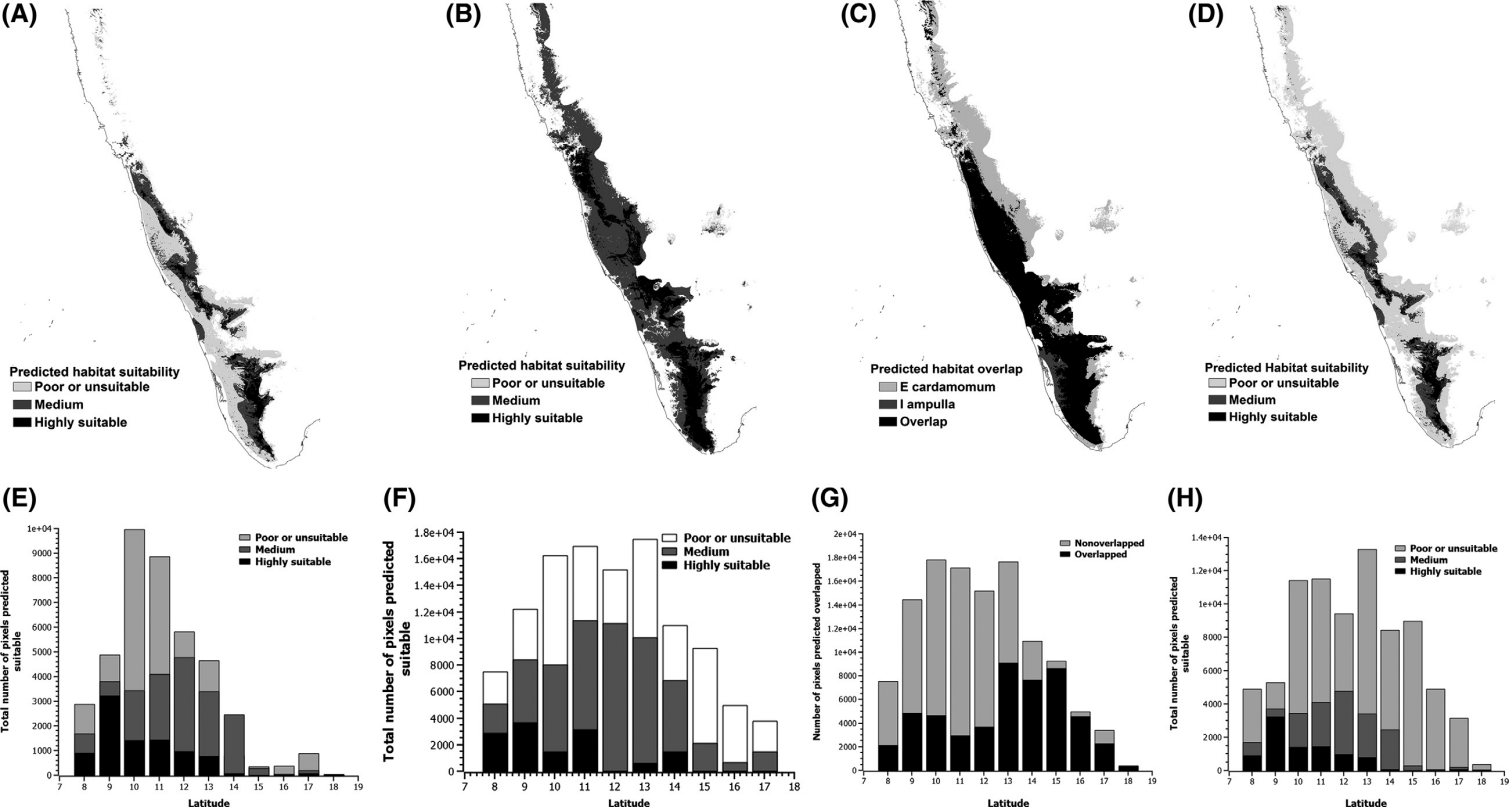
The maps and bar graphs of distribution of suitable habitats and habitat overlap across latitude for *Indrella ampulla* and *Ellettaria cardamomum* in Western Ghats for the present climate scenario. (A) Habitat suitability map of *I. ampulla*, (B) habitat suitability map for *E. cardamomum* cultivation, (C) map showing potential habitat overlap between *I. ampulla* and *E. cardamomum*, (D) map showing differential suitability of *E. cardamomum* cultivation areas to *I. ampulla* distribution, (E) distribution of differentially suitable habitat for *I. ampulla* across latitude, (F) distribution of differentially suitable habitat for *E. cardamomum* cultivation across latitude, (G) distribution of potentially overlapped habitat for *I. ampulla* and *E. cardamomum* across latitude, and (H) distribution of *E. cardamomum* cultivation areas differentially suitable for *I. ampulla* across latitude.

The suitable habitats for both *I. ampulla* and *E. cardamomum* were found in southern part of Western Ghats (8°N to 12°N) followed by central part of Western Ghats (13°N to 14°N) and decreased toward northern part of Western Ghats (15°N to 18°N) (Fig. 2A, B, E, and F). In total, 72,271 km^2^ (45.17% of Western Ghats) of area for *I. ampulla* and 114,323 km^2^ (71.45% of Western Ghats) area for *E. cardamomum* were found suitable. However, not all areas in the Western Ghats region are uniformly suitable either for *I. ampulla* or for *E. cardamomum*. There were distinct patches in the Western Ghats that were predicted to be highly suitable (dark black regions (39,378 km^2^ for *I. ampulla* and 13,164 km^2^ for *E. cardamomum*)) as opposed to several patches that are poor or not suitable (light gray to white) regions covering 8820 km^2^ for *I. ampulla* and 61,943 km^2^ for *E. cardamomum* (Fig. 2A, B, E, and F). For both species, highly suitable sites were located in southern Western Ghats (8°N to 12°N) followed by central Western Ghats (13°N to 14°N), and the majority of the land area in northern part were poorly suitable for both species (15°N to 18°N) (Fig. 2A, B, E, and F).

Comparisons between two general circulation models (CSIRO and HADCM3) under both climate change scenarios (B1 and A2) for two species showed a decreasing trend in suitable habitat by 2080 as compared to the present (Appendix S6, Figs S5A–H and S6A–H). Since the predicted results of both models were congruent, we combined models separately for two climatic scenarios. In both species, the A2 scenario presented a higher percentage of loss than B1 (Appendix S6, Figs S5A–H and S6A–H). Although the percentage of current range was maintained same under both scenarios (Appendix S6, Figs S5A–H and S6A–H), most of it was poorly suitable for the species by 2080. Moreover, for both species, most of their current habitats that were predicted as highly suitable will become poorly suitable or unsuitable by 2080 (Appendix S6, Figs S5A–H and S6A–H).

### Combining predicted habitat suitability and demographic parameters

**Figure 3:**
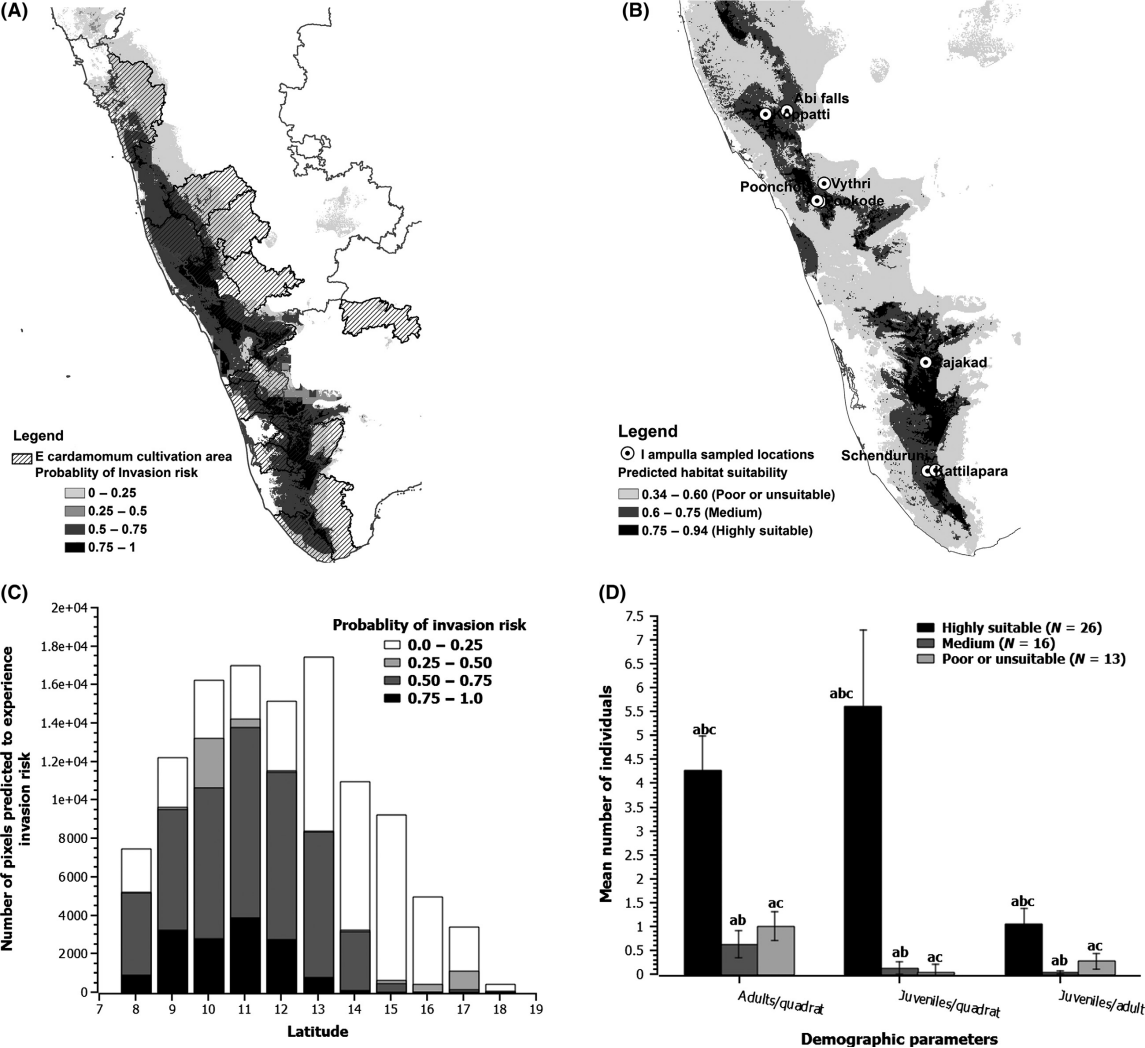
Map of invasion risk and sampled locations of demographic data of *Indrella ampulla*. Bar graphs depict the probability of invasion risk across latitude and demographic parameters of *I. ampulla* across different habitat suitability in Western Ghats. (A) Map showing probability of invasion risk of *I. ampulla* to *Ellettaria cardamomum* cultivation areas in Western Ghats, (B) map showing sampled populations of *I. ampulla* with their respective habitat suitability category to collect demographic data, (C) bar graph showing the probability of invasion risk of *I. ampulla* to *E. cardamomum* cultivation areas across latitude in Western Ghats, and (D) bar graph showing mean variation in demographic parameters of *I. ampulla* across different habitat suitability. *Note*: The invasion risk map is overlapped with layer of *E. cardamomum* cultivation areas in Western Ghats. The bars above the graph indicate standard error of mean for each demographic parameter. The dissimilar letters above the bar indicates that *t*‐test significant at *P* < 0.05.

The models sufficiently discriminated and assigned sites selected for the demographic data collection into three categories of habitat based on their habitat suitability values and the chosen threshold value (Fig. 3B and Appendix S5, Table S5). As per the predictions, the populations of *I. ampulla* located in highly suitable habitat had high density of adults (*t*‐test, *P* < 0.05) and juveniles (*t*‐test, *P* < 0.05) as compared to poorly suitable habitats (Fig. 3D). Moreover, the populations from highly suitable habitat also had highest number of juveniles per adult as compared to populations from poorly suitable habitats (*t*‐test, *P* < 0.05), indicating individuals of *I. ampulla* from highly suitable habitat had higher reproductive rate as compared to individuals from poorly suitable habitats (Fig. 3D). The site (Rajakkad in Idukky district of Kerala region) that had highest habitat suitability values was found to have higher density of adults and juveniles and also more number of juveniles per adult than other sites (Fig. 3B and Appendix S5, Table S5). Interestingly, the first reports of *I. ampulla* as a pest (Sudhi 2010) have been reported from the site (Rajakkad), which also exhibited highest habitat suitability values in our model.

### Prediction of habitat overlap and invasion risks

The overlap between suitable habitats of *I. ampulla* and *E. cardamomum* was high, and 76.4% of habitats suitable for *I. ampulla* overlapped with habitats of *E. cardamomum* (Fig. 3C). The overlap was high in the central (13–14°N) and northern (15–18°N) part of Western Ghats as compared to southern region (8–12°N) (Fig. 2G). However, not all areas of *I. ampulla* that overlapped with the suitable habitats of *E. cardamomum* were uniformly suitable for *I. ampulla*. There were distinct patches in the overlapped areas that were predicted to be highly suitable (dark black regions) as opposed to certain patches that are poor or not suitable (light gray to white regions) (Fig. 2D). The suitable habitat for *E. cardamomum* cultivation in southern Western Ghats (8–12°N) had highest overlap with highly suitable habitat of *I. ampulla* (Fig. 2H), and the suitable habitat for *E. cardamomum* cultivation in central (13–14°N) and northern (15–18°N) Western Ghats mostly overlapped with poorly suitable habitat of *I. ampulla* (Fig. 2H).

**Figure 4:**
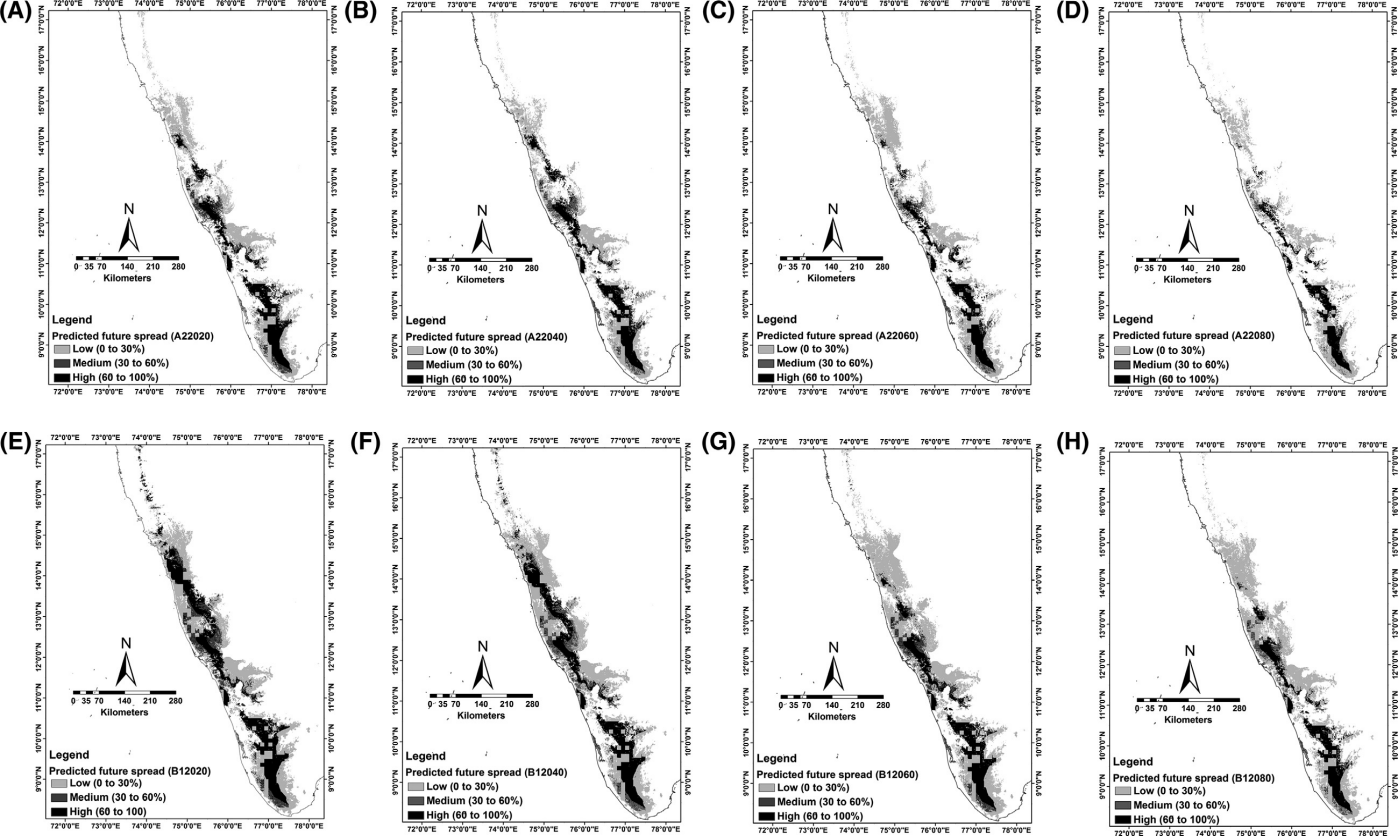
Map of future invasion risk of *Indrella ampulla* to *Ellettaria cardamomum* cultivation areas in Western Ghats modeled using constrained dispersal using MIGCLIM and Maxent under the assumption of future climate (2020–2080) scenario. (A) 2020 A2, (B) 2040 A2, (C) 2060 A2, (D) 2080 A2, (E) 2020 B1, (F) 2040 B1, (G) 2060 B1, (H) 2080 B1. *Note:* The maps of invasion risk for future climate scenario represent the consensus of two general circulation models (CSIRO_MK3 and UKMO_HADCM3) for two SRES emission scenarios (B1 and A2). The maps were rescaled to % probability map to identify *E. cardamomum* cultivation areas highly sensitive to *I. ampulla* invasion using the maximum training sensitivity plus specificity (MTSS) threshold. The dark black shades indicate *E. cardamomum* cultivation areas highly sensitive to spread of *I. ampulla* in Western Ghats.

It is predicted that under the future climatic scenario, the overlap between suitable habitat of *I. ampulla* and suitable habitat for *E. cardamomum* cultivation will decrease (Appendix S6, Fig. S7A–H). The A2 scenario presented a more drastic decrease in overlap between suitable habitats of *I. ampulla* and suitable habitats of *E cardamomum* cultivation than the B1 scenario (Fig. 4A and H).

The invasion risk model identified that *E. cardamomum* cultivation areas in southern Western Ghats are highly vulnerable to *I. ampulla* invasion (8–12°N) (Fig. 3A and C), and the vulnerability of *E. cardamomum* cultivation areas to *I. ampulla* invasion decreases toward central (13–14°N) and northern (13–14°N) part of Western Ghats (Fig. 3A and C). Interestingly, the cardamom cultivation areas near to the sites where high density of adults and juveniles of *I. ampulla* reported is predicted to be in highly vulnerable zone for *I. ampulla* invasion (Fig. 3A and B).

The invasion risk model of *I. ampulla* under future climatic scenario indicated that the *E. cardamomum* cultivation areas in southern part (8–12°N) of Western Ghats continues to experience the spread of *I. ampulla* (Fig. 4A–H). Moreover, some areas in central (13–14°N) and northern parts (15–19°N) of Western Ghats were also predicted to be susceptible for *I. ampulla* invasion (Fig. 4A–H). However, these areas are predicted to become unsuitable for both *I. ampulla* distribution and *E. cardamomum* cultivation by the end of 2080 (Fig. 4A–H), and invasion of *I. ampulla* to these areas is predicted to decrease. The probability of spread was predicted high for B1 scenario than A2 scenario and decreased with time (Fig. 4A–H).

## Discussion

### Model calibration and important predictors of habitat suitability

We found improved model performance for *I. ampulla* after accounting for sampling bias as compared to earlier models highlighting the importance of accounting for sampling bias in modeling species distribution as per Elith et al. (2010). In case of *E. cardamomum*, the model was calibrated using both wild and cultivated range records and accounting for sampling bias. Mau‐Crimmins et al. (2006) and Beaumont et al. (2009) evaluated the importance of calibrating models using either native range data (wild) or invaded range data. Models tend to perform better when calibrated using invaded range data than native (wild) range data (Mau‐Crimmins et al. 2006; Beaumont et al. 2009). Models calibrated using invaded range data can consider factors not present in the native range, such as environmental preferences of the invaded range genotype (Beaumont et al. 2009). The results of *E. cardamomum* model calibration using wild and cultivated range data contradict the results of (Beaumont et al. 2009), and we did not find significant difference in model performance among models calibrated using either wild range data or cultivated range data or not even accounting for sampling bias toward cultivated and wild range data (for all the treatments, AUC was >0.9). It is probably because the *E. cardamomum* is native to Western Ghats, and most of the cardamom cultivation areas are located in its native range Western Ghats. As a result the environmental preferences for both wild and cultivated *E. cardamomum* genotypes, may not differ much. However, the model calibrated using all cultivated records and 50% of wild records with accounting sampling bias for cultivated records produced best model of habitat suitability for *E. cardamomum* cultivation in Western Ghats than other treatments. Including both cultivated and wild records can model the suitable areas for cultivation of native crops domesticated in its native range alone using either wild or cultivated records.

Our results indicate that among 18 environmental variables used, two climatic variables (Bioclim19: precipitation of coldest quarter and Bioclim 3: isothermality, which is a measure of day and night temperature oscillation) along with the edaphic variable cation exchange capacity (CEC) of soil are the best predictors of *I. ampulla* distribution in Western Ghats. Similar climatic variables have been reported in influencing distribution and range expansion of other invasive gastropods (McDowell 2014). On the other hand, CEC of soil, which is a measure of exchangeable cations (Na^+^, K^+^, Ca^+^, and Mg^+^) and aluminum (Al) content in soil, is also reported as important factors for the distribution of land snails (Outeiro 1988; Riballo 1990; Outeiro et al. 1993). Our results indicated that aridity (which is a measure of humidity) and elevation were also important factors in predicting potential distribution of *I. ampulla* in Western Ghats. These variables were also reported as important factors affecting distribution of gastropods (Cameron 1973; Nekola 2003). In summary, our results suggest that the distribution of highly suitable habitats for *I. ampulla* was mainly located in highly humid and high elevation (1800–2100 m) areas with high precipitation in warmest quarter (>5000 mm), high isothermally (63–66), and lower topsoil CEC (0–5 cmol/kg of soil) (Appendix S3, Fig. S4A–E).

However, there may be other abiotic and biotic variable, which influence distribution and spread of *I. ampulla* to *E. cardamomum* plantations in Western Ghats. *I. ampulla* prefers bioclimatically stable habitat such as rain forests of Western Ghats. Nevertheless, due to growing demands of human population, most of its habitat has been taken over by coffee and cardamom planters. Interestingly, most of the places where *Indrella* is distributed are also very suitable for growing cardamom. However, our results also confirm that the same environmental variables, which are predicted to be important for *I. ampulla* distribution, were also major predictors of potential suitable areas of *E. cardamomum* cultivation in Western Ghats (Table 2, Appendix S3, Figs S3A–E and S4A–E). Both the species require similar environmental conditions, which could be the reason for our frequent encounters of many populations of I. ampulla in cardamom plantations near to the forest.

Our results indicate that the population density of *Indrella* was positively influenced by canopy cover and litter depth (Table S3). The reduction of canopy cover in natural forests as a result of its conversion to cardamom plantations, many *Indrella* populations were able to spread to these plantations. Excessive calcium supply by cardamom planters to control blight diseases and reduce soil acidity is also reason the snail prefers to disperse into the cardamom plantation in addition to the aforementioned reasons. Calcium is very important for physiological process, reproduction, and most notably shell production of snail (Fournié & Chetail 1984; Burch & Pearce 1990). Hence, the snails might have spread to the cardamom plantations, where calcium supply is high and is easily available compared to natural forest where snails have to obtain calcium by ingesting calcium particle from the soil, rasping calcium rich rocks, and digesting decaying leaf matter (Fournié & Chetail 1984; Burch & Pearce 1990; Wäreborn 1979, 1992). Our results also confirmed that cation exchange capacity (CEC) and calcium carbonate (CaCO3) content of top soil are very important for *I. ampulla* distribution (Table 2).

### Predicted habitat suitability and demographic parameters

The majority of the suitable habitat for both *I. ampulla* and *E. cardamomum* were found in southern Western Ghats. However, most of the highly suitable habitats of *I. ampulla* will become poor or unsuitable for the species by 2080 (Appendix S6, Fig. S5A–H), and many of the present *E. cardamomum* growing areas in Western Ghats are predicted to become unsuitable for Cardamom cultivation in future under the scenario A2 (Appendix S6, Fig. S6A–H). Overall, the results clearly indicate that the future climate may negatively impact both *I. ampulla* distribution and *E. cardamomum* cultivation areas in Western Ghats.

Combining habitat suitability model predictions with demographic parameters indicated that the habitat suitability might play a major role in establishment success and potential invasion of *I. ampulla* to *E. cardamomum* plantations in Western Ghats. Our results indicated that the cardamom plantations that overlapped with highly suitable habitat of *I. ampulla* had high density of adults and juveniles of *I. ampulla* and thus higher reproductive potential. For example, the cardamom plantation from Rajakkad located in Kerala state (southern Western Ghats) had highest density of adults and juveniles of *I. ampulla* and also had higher reproductive potential. Our model predicted that this location overlaps with one of the highly suitable habitats of *I. ampulla* with highest habitat suitability values (Table S5). Moreover, the *I. ampulla* individuals collected from this region exhibited highest egg laying capacity (the two individuals collected and monitored in captivity laid 130 eggs together) as compared to individuals collected from Koppati, Coorg (two individuals together laid 35 eggs). Indicating *I. ampulla* populations located in Rajakkad had highest fecundity rate and have potential to become a pest in this region. Interestingly, from the same location (Sudhi 2010) I. ampulla has been reported as pest.

### Predicted habitat overlap and invasion risk

There was a high degree of overlap between suitable habitat of *I. ampulla* and predicted suitable areas for *E. cardamomum* cultivation under both present and future climatic scenarios. This pattern is expected because similar environmental variables limit the distribution of both *I. ampulla* and *E. cardamomum* (Table 2). However, not all overlapped habitats of *I. ampulla* with *E. cardamomum* cultivation areas were uniformly suitable for *I. ampulla* distribution. The overlapped habitat varied from those that are highly suitable to poor or unsuitable for *I. ampulla* distribution. The majority of overlapped highly suitable habitat for *I. ampulla* was located in southern Western Ghats, indicating that *E. cardamomum* cultivations areas located in the southern region are highly vulnerable to *I. ampulla* invasion. Moreover, the maps of habitat overlap for future climatic scenario (2020–2080) indicated that *E. cardamomum* cultivation areas predicted to overlap with *I. ampulla* habitat in central and northern part of Western Ghats decrease by 2080 (Appendix S6, Fig. S6A–H) under both A2 and B1 climate change scenarios. However, *E. cardamomum* cultivation areas in the southern Western Ghats were predicted to overlap continuously over this period of time (2020–2080). This suggests that the *E. cardamomum* cultivation areas in southern Western Ghats are highly susceptible to the spread of *I. ampulla* under future climate scenarios. The prediction of invasion risk model suggests that in the absence of appropriate management strategies, *I. ampulla* could spread from its current distribution across a large portion of *E. cardamomum* cultivation areas in Western Ghats. The predicted distribution maps indicate that the distribution of *I. ampulla* is primarily based on environmental suitability. In order to understand overall invasion risk, additional factors need to be considered (Richardson and van Wilgen 2004). Our model for *I. ampulla* invasion risk under the present and future climate scenario incorporates environmental suitability for *I. ampulla*, distance of predicted *E. cardamomum* cultivation areas to *I. ampulla* occurrence records, transformed land cover data (tropical broadleaved or rain forest), and dispersal constrains of *I. ampulla*. Invasion risk is likely to be highest in *E. cardamomum* cultivation areas that overlap with habitats with highly suitable environmental conditions for *I. ampulla*, which are near the tropical rain forest and are in close proximity to source of *I. ampulla* propagules.

Based on the invasion risk model, many *E. cardamomum* cultivation areas in Western Ghats are subject to high risk of *I. ampulla* invasion. Some of the *E. cardamomum* cultivation areas in the central and northern Western Ghats identified as environmentally very suitable for *I. ampulla* that occur near or within tropical rain forests are separated by a large distances from source propagules of *I. ampulla* (Fig. 3A). Although these *E. cardamomum* cultivation areas are considered as highly suitable for *I. ampulla*, they are not necessarily at high risk of invasion in the immediate future. However, our model indicates that many *E. cardamomum* cultivation areas in southern Western Ghats (mainly 9°N to 10°N) are at immediate risk. Because they are near to sources of *I. ampulla* propagules, for example, *E. cardamomum* cultivation sites near to Rajakkad where *I. ampulla* is reported as a pest due to its higher population density and higher propagule production. Our future invasion risk maps (2020–2080) also indicated that *E. cardamomum* cultivation areas in southern Western Ghats are under high invasion risk as compared to central and northern parts, and they are predicted to continuously experience the spread of *I. ampulla* through 2020–2080 (Fig. 4A–H).

Our analysis indicates that there are many *E. cardamomum* cultivation areas are at invasion risk, but have not been invaded by *I. ampulla*. This provides an opportunity to implement management strategies to prevent invasion of *I. ampulla* to *E. cardamomum* plantations. However, our field visits and communication with cardamom growers revealed that *I. ampulla* can be easily dispersed through organic manure transported from *I. ampulla* infested sites and may hamper efforts to prevent *I. ampulla* invasion to other *E. cardamomum* cultivation areas.

### Conservation and management implications

Through inclusion of multiple factors including environmental suitability, demographic parameters that are reported to be important for establishment success of species, availability of propagule source (distance map), land cover data, and dispersal constrains of target species, we provide effective yet simple model of *I. ampulla* invasion risk to *E. cardamomum* cultivation areas in Western Ghats under both the present and future climate scenarios. Our study also identifies factors associated with the distribution and successful invasion of *I. ampulla* to *E. cardamomum* cultivation areas in Western Ghats. Our invasion risk model suggests that *I. ampulla* will continue to spread to other *E. cardamomum* cultivation areas in Western Ghats. We also provide future maps of environmental suitability and habitat overlap for *I. ampulla* and *E. cardamomum* in Western Ghats and identify possible areas of future invasion by *I. ampulla*. This information is useful for cardamom planters, managers, and policy makers to design effective management strategies to control further spread of *I. ampulla* to *E. cardamomum* plantations in Western Ghats. However, land use practices including cropland areas are not static (Foley et al. 2005), and many of cardamom cultivation areas may not have *I. ampulla* or those areas may not be suitable for *I. ampulla* distribution. Moreover, *I. ampulla* is an endemic species and has not been reported as pest throughout its native range except for few locations where their populations have drastically increased in the recent years. Thus, we suggest that our predictions can be used as a dynamic tool to decide where it is necessary to concentrate on monitoring and implement strategies to prevent *I. ampulla* invasion to *E. cardamomum* plantations in Western Ghats.

One of the greatest challenges for preventing colonization and spread of *I. ampulla* to cardamom plantations is the spread of the species through human‐mediated dispersal by transport of manures. Since snails are poor dispersers (Aubry et al. 2006), natural dispersal is of minor importance. During our field study, cardamom planters in Kerala (southern Western Ghats) indicated snails getting dispersed through manure is common. Thus, the recent invasion of *I. ampulla* to many cardamom plantations in southern Western Ghats could be through the transport of manures from *I. ampulla* infested sites. Thus, our maps are useful for cardamom planters to identify locations, which are free of *I. ampulla* occurrence to import organic manures and avoid accidental introductions to unaffected areas. Our results are invaluable in detecting the direction of potential spread and places where invasion of *I. ampulla* to *E. cardamomum* cultivation areas occurs. Our results suggest that *I. ampulla* may spread from southern Western Ghats, which is identified as hot spot of *I. ampulla* invasion to *E. cardamom* cultivation areas in central and northern Western Ghats. *Indrella ampulla* is considered as a monotypic genus, which exhibit color polymorphisms in their soft body parts. It is also worthy to mention that the yellow color morph of the same species is not yet reported as a pest for cardamom, eventhough their distribution overlaps in areas such as Wayand in Central Western Ghats. Similarly, the red morphs are emerging as a pest in Idukky regions and has not yet been reported as a pest from other parts of its range. These information are vital, which needs to be considered a priori while designing management strategies.

In conclusion, the prediction from ecological niche models along with other data including demographic data, proximity of crop areas to pest or invasive species propagules, land cover data, and integrating dispersal constrains of the invasive or native pest organisms serve as a useful tool to identify factors and process influencing potential invasion of pest species. This information is valuable for identifying suitable cardamom cultivation areas avoiding vulnerability to the pest invasions under the present and future climate scenario. Moreover, the predictions and results obtained from these tools should be incorporated into integrated pest management schemes to prevent the spread of native and invasive pest species.

## Conflict of Interest

None declared.

## Data Accessability

All data used in this article will be available in Dryad Digital Repository (DOI: doi:10.5061/dryad.bp683)

## Supporting information


**Appendix S1.** Occurrence records and predicting habitat suitability for *I. ampulla* and *E. cardamomum* in present and future climate scenario.
**Table S1.** The details of variables used to predict the potential distribution of *I. ampulla* and *E. cardamomum*.
**Figure S1.** The distribution maps of *I. ampulla* and *E. cardamomum* and adult and juvenile of *I. ampulla* (A) distribution map of *I. ampulla* (B) distribution map of *E. cardamomum* (C) different color morphs of *I. ampulla* and (D) juvenile of *I. ampulla (red morph)*.
**Table S2.** The different treatment and number of occurrence records used to calibrate the *E. cardamomum* model.
**Appendix S2**. Detailed Methodology of creating bias file for *Indrella ampulla* and *E. cardamomum*.
**Figure S2.** Bias grids created for *I. ampulla* and *E. cardamomum* (A) bias grid of *I. ampulla* (B) bias grid of *E. cardamomum* created using all (both cultivated and wild) records (C) bias grid of *E. cardamomum* created using only cultivated records (D) bias grid of *E. cardamomum* created using only wild records (see Appendix S1 for how these bias grids were created).
**Appendix S3**. Results and discussion of Important predictors of habitat suitability for *I. ampulla* and *E. cardamomum*.
**Table S3.** Relationship of snail density with microhabitat parameters and extant of disturbance noticed.
**Figure S3.** Marginal response curves of the predicted probability of suitable habitat of *E. cardamomum* in Western Ghats for five environmental variables that contributed substantially to the MaxEnt model and jackknife analysis of individual predictor environmental variables important in the development of full habitat suitability model for *E. cardamomum* (A) precipitation of coldest quarter (B) isothermality (C) maximum temperature warmest month (D) elevation (E) Aridity index (F) training gain for environmental variables (G) test gain for environmental variables and (H) test AUC for environmental variables.
**Figure S4.** Marginal response curves of the predicted probability of suitable habitat of *I. ampulla* in Western Ghats for five environmental variables that contributed substantially to the MaxEnt model and in the jackknife analysis of individual predictor environmental variables important in the development of full habitat suitability model for *I. ampulla* (A) precipitation of coldest quarter (B) isothermality (C) topsoil cation exchange capacity (D) elevation (E) Aridity index (F) training gain for environmental variables (G) test gain for environmental variables and (H) test AUC for environmental variables.
**Appendix S4.** Result and discussion for Model calibration of *E. cardamomum*.
**Table S4.** The area under curve (AUC) values and average similarity of different treatments used to calibrate the *E. cardamomum* model.
**Appendix S5.** Detailed methodology and discussion of combining predicted habitat suitability and demographic parameters of *I. ampulla*.
**Table S5.** The eight *I. ampulla* populations chosen for collecting demographic data with their respective habitat suitability, No of quadrats surveyed and demographic parameters.
**Appendix S6.** Detailed methodology for predicted habitat overlap for *I. ampulla* and *E. cardamomum* in future climate scenario.
**Figure S5**. Distribution of available suitable habitats for *I. ampulla* in Western Ghats modelled using MaxEnt under the assumption of future climate (2020–2080) scenario.
**Figure S6.** Distribution of available suitable habitat for *E. cardamomum* cultivation in Western Ghats modelled using MaxEnt under the assumption of future climate (2020–2080) scenario.
**Figure S7.** The map of potential habitat overlap of *E. cardamomum* cultivation areas and *I. ampulla* in Western Ghats as modelled by MaxEnt under the assumption of future climate (2020–2080) scenario.
**Figure S8.** Habitat suitability and overlap predictions for *I. ampulla* and *E. cardamomum* for the period 2080 using RCP scenario 2.6 and 8.5.
