# Supplementary material for: Ecological niche modeling for conservation planning of an endemic snail in the verge of becoming a pest in cardamom plantations in the Western Ghats biodiversity hotspot
Source: Ecol Evol. 2016 Aug 18;6(18):6510–23. doi: 10.1002/ece3.2368 (PMC5058523; doi:10.1002/ece3.2368)
Supplement: Supplementary file 1 — Appendix S1. Occurrence records and predicting habitat suitability for I. ampulla and E. cardamomum in present and future climate scenario. Table S1. The details of variables used to predict the potential distribution of I. ampulla and E. cardamomum. Figure S1. The distribution maps of I. ampulla and E. cardamomum and adult and juvenile of I. ampulla (A) distribution map of I. ampulla (B) distribution map of E. cardamomum (C) different color morphs of I. ampulla and (D) juvenile of I. ampulla (red morph). Table S2. The different treatment and number of occurrence records used to calibrate the E. cardamomum model. Appendix S2. Detailed Methodology of creating bias file for Indrella ampulla and E. cardamomum. Figure S2. Bias grids created for I. ampulla and E. cardamomum (A) bias grid of I. ampulla (B) bias grid of E. cardamomum created using all (both cultivated and wild) records (C) bias grid of E. cardamomum created using only cultivated records (D) bias grid of E. cardamomum created using only wild records (see Appendix S1 for how these bias grids were created). Appendix S3. Results and discussion of Important predictors of habitat suitability for I. ampulla and E. cardamomum. Table S3. Relationship of snail density with microhabitat parameters and extant of disturbance noticed. Figure S3. Marginal response curves of the predicted probability of suitable habitat of E. cardamomum in Western Ghats for five environmental variables that contributed substantially to the MaxEnt model and jackknife analysis of individual predictor environmental variables important in the development of full habitat suitability model for E. cardamomum (A) precipitation of coldest quarter (B) isothermality (C) maximum temperature warmest month (D) elevation (E) Aridity index (F) training gain for environmental variables (G) test gain for environmental variables and (H) test AUC for environmental variables. Figure S4. Marginal response curves of the predicted probability of suitable habitat [file ECE3-6-6510-s001.doc]

**Supporting Information Appendix S1 to S6**

**Ecological niche modelling for conservation planning of an endemic snail in the verge of becoming a pest in cardamom plantations in the Western Ghats biodiversity hotspot**

**Appendix S1. Occurrence records and predicting habitat suitability for *I. ampulla* and *E. cardamomum* in present and future climate scenario.**

*Occurrence records*: The records for *I. ampulla* were obtained from online database (http://indiabiodiversity.org/species) and field survey undertaken over a two year period (2009 to 2011) in the Western Ghats. The records for .*E cardamomum* included both wild and cultivated locations and they were derived from several sources including online database (<http://indiabiodiversity.org/species>), literature (Kuriakose *et al*. 2009; Kumar *et al*.1995; Prasath *et al*. 2004) and field survey (2009 to 2011) undertaken in Western Ghats.

Maps of occurrence data for the both species were produced to check for oblivious errors such as outlying localities (points outside the geographic distribution of species) and pseudo-replicates (Fig S1a and S1b). To avoid pseudo-replication only one occurrence record per 1 km grid cell was used for model prediction and outlying localities were removed. In total, we obtained 32 unique records for *I. ampulla* and 67 unique records (37 cultivated and 30 wild) for *E cardamomum* in Western Ghats. ArcGIS 10 (ESRI 2012) was used for all mapping outputs.

For *I. ampulla,* habitat suitability model was developed by using 18 of the chosen 21 variables (Table S1). We removed three variables (T_BD, PET and AET (Table 1) because they were not biologically relevant for *I. ampulla* distribution. In the case of *E. cardamomum*, we used all 21 environmental variables to model the areas suitable for cardamom cultivation in Western Ghats. As *E. cardamomum* records were collected from both cultivated and wild locations, we calibrated *E. cardamomum* habitat suitability model with 15 different treatments, which is having 5 different set of occurrence records multiplied by 3 different types of bias files (Table S2 and Fig. S2b to S2d). We used these 15 different treatments to account for spatial sampling bias of cultivated and wild range of species and to predict unbiased model of suitable habitat for cardamom cultivation in Western Ghats. The same MaxEnt settings to that of *I. ampulla* were used in all 15 different treatments to model the suitable habitat. Finally, a single model with best prediction of habitat suitability for *E. cardamomum* in Western Ghats was chosen by calculating pairwise similarity between the models obtained from each 15 different treatment and average consensus model of all 15 different treatments (Table S4). The treatment model that was having highest average similarity was chosen for further analysis and to use in future climatic modelling (Table S4). The similarity analysis was done in ArcMap, ArcGIS 10.0.

The future climate data downscaled using the delta method to 30 arc seconds (1000m) was used to predict the suitable habitat for *I. ampulla* and *E. cardamomum* in future climate scenario. The delta method is the interpolation of General Circulation Model generally used in climate modelling at scales of 100 to 200 km using a thin plate spline spatial interpolation method to achieve the 30 arc seconds resolution . The data was provided by the CGIAR Research Program on Climate Change, Agriculture and Food Security (CCAFS) (http:// [www.ccafs-climate.org/statistical downscaling_delta/](http://www.ccafs-climate.org/statistical downscaling_delta/)) (Ramirez & Jarvis 2008). We projected future climate for four time period starting from 2020 to 2080 with 20 year interval (2020, 2040, 2060 and 2080) using two different general circulation models (GCM) such as Hadley Centre Coupled Model, version 3 HadCM3 (Gordon et al.2000) and Commonwealth Scientific and Industrial Research Organization (CSIRO), for two different SRES (Beaumont et al. 2009) emission scenarios: A1, which includes large human population growth, large amounts of energy use and slow technological change; B2 scenario, which includes medium population growth and a mix of energy sources that are consistent with usage today (IPCC 2000). There are several climate models available (e.g.HadCM3, CCCMA and CSIRO) but the HadCM3 and CSIRO model was selected for this study because, it is reported to provide better mean results for Asia compared with other models (Christensen *et al*. 2007). Finally, we included topographic and edaphic layers as static variables with projected climate (bioclim) layers (dynamic variables) (Table S1). It has been recently shown that, including static variables in the future model with the dynamic variables could perform better or no worse than excluding the static variables (Stanton et al. 2011). To compare the models in a temporal series, we used an ensemble–consensus approach (Araújo & New 2006). The calculation of potential effects of climate change was based on the variation (2020 to 2080) that could be found in each pixel with respect to the present prediction, generating different comparisons considering separately two emission scenarios (A2 and B1). The assessment of the variation of models in relation to the current range was analyzed by the range lost and the range gained, hypothesizing that both species could reach new potential sites. Visualization of the resulting maps was done using ArcGIS 10.0 (ESRI 2012).

An important step in evaluating the model performance is to verify that the data used to train and test the model performed significantly better than the random. The model performance was evaluated using two commonly used validation indices; the AUC (Area under the curve), and the True Skill Statistic (TSS). The AUC validation statistic is a commonly used threshold independent accuracy index that ranges from 0.5 (not different from a randomly selected predictive distribution) to 1 (with perfect predictive ability). Models having AUC values >0.9 were considered to have very good, >0.8 good and >0.7 useful discrimination abilities (16). AUC also reflects the model's ability to distinguish between presence records and random background points. The TSS statistic ranges from −1 to +1 and tests the agreement between the expected and observed distribution, and whether that outcome would be predicted under chance alone (Allouche *et al*. 2006; Liu *et al*. 2009). A TSS value of +1 is considered perfect agreement between the observed and expected distributions, whereas a value <0 defines a model which has a predictive performance no better than random (Allouche *et al*. 2006). TSS was shown to produce the most accurate predictions (Jiménez-Valverde *et al*. 2004).

The habitat suitability for both species in present and future climate scenario in Western Ghats was modeled with default settings in MaxEnt except for following changes: Random test percentage was set to 30%. Regularization multiplier was set to 1 and maximum number of background points for sampling was kept at 10,000 (see Appendix S2 for a full description of the methodology used for background point selection). Some parts of Western Ghats have been more intensively sampled for species than others. In order to control for this sampling bias (Phillips et al. 2009), we used bias files (Fig. S2a to d) in MaxEnt modeling (see Appendix S2, for a full description of the methodology used to create bias file).

We ran 50 replicates for the species and averaged the results. Maximum iterations were set to 5000, with 1*10^6^ convergence threshold. Auto feature of environmental variables was selected. A 50-fold cross-validation was used to test model performance of species. Jackknife procedure and percent variable contributions was used to estimate the environmental variable influence on each species.

The logistic output was chosen to generate the model and the result of the Maxent model output was the continuous probability of the occurrence value ranging from 0.0 to 1.0, where higher values means better suitability and vice versa. We transformed the predicted continuous values into a categorical prediction (i.e, highly suitable, medium and poor or unsuitable). The logistic threshold at maximum training sensitivity plus specificity was used for categorical classification (Table 1) as this approach is considered as one of the promising approaches for predicting species distributions (Liu *et al*. 2005; Cuest-Comocho *et al*. 2006).

**Table S1:** The details of variables used to predict the potential distribution of *I. ampulla* and *E. cardamomum*.

| **Layer** | **Reference** | **Variables** |
| --- | --- | --- |
| **Bioclimatic** | | |
| **Bioclim1** | <http://www.worldclim.org/current>)  (Current)  (<http://www.ccafs-climate.org/data>)  (Future) | Annual Mean Temperature (°C*10) |
| Bioclim2 |  | Mean Diurnal Range (Mean (period max-min)) (°C*10) |
| Bioclim3 |  | Isothermality (Bioclim2/Bioclim7) (°C*10) |
| **Bioclim4** |  | Temperature Seasonality (SD*100) |
| Bioclim5 |  | Max Temperature of Warmest month (°C*10) |
| Bioclim6 |  | Min Temperature of Coldest month (°C*10) |
| **Bioclim7** |  | Temperature Annual Range (Bioclim5-Bioclim6) |
| **Bioclim8** |  | Mean Temperature of Wettest Quarter (°C*10) |
| **Bioclim9** |  | Mean Temperature of Driest Quarter (°C*10) |
| **Bioclim10** |  | Mean Temperature of Warmest Quarter (°C*10) |
| **Bioclim11** |  | Mean Temperature of Coldest Quarter (°C*10) |
| **Bioclim12** |  | Annual Precipitation (mm) |
| **Bioclim13** |  | Precipitation of Wettest Period (mm) |
| **Bioclim14** |  | Precipitation of Driest Period (mm) |
| Bioclim15 |  | Precipitation Seasonality (Coefficient of Variation) |
| Bioclim16 |  | Precipitation of Wettest Quarter (mm) |
| Bioclim17 |  | Precipitation of Driest Quarter (mm) |
| Bioclim18 |  | Precipitation of Warmest Quarter (mm) |
| Bioclim19 |  | Precipitation of coldest Quarter (mm) |
| **Global aridity and Evapo-Transpiration** | | |
| AET | <http://www.cgiar-csi.org/data/global-aridity-and-pet-database>) | Annual evapo-transpiration |
| PET |  | Potential evapo-transpiration |
| AI |  | Global aridity index |
| **Edaphic** | | |
| T_OC | <http://webarchive.iiasa.ac.at/Research/LUC/External-World-soil-database>) | Topsoil total organic carbon |
| T_CaCO3 |  | Topsoil Calcium carbonate |
| T_CEC |  | Topsoil cation exchange capacity |
| T_PH_H2O |  | Topsoil PH(H_2_O) |
| T_ECE |  | Topsoil salinity |
| T_BD |  | Topsoil Bulk density |
| **T_TBS** |  | Topsoil total exchangeable bases |
| **Topographic** | | |
| DEM | <https://lta.cr.usgs.gov/gtopo30>) | Digital Elevation model |
| Aspect | <https://lta.cr.usgs.gov/HYDRO1K>) | Direction of slope |
| Slope |  | Difference between two neighboring cells elevation |

Note: The collinear variables with a Pearson’s correlation coefficient ≥ 0.7 (r^2^ > 0.49) is represented in bold

**Figure S1.** The distribution maps of *I ampulla* and *E cardamomum* and adult and juvenile of *I. ampulla* a) distribution map of *I. ampulla* b) distribution map of *E. cardamomum* c) different color morphs of *I. ampulla* and d) juvenile of *I. ampulla*.


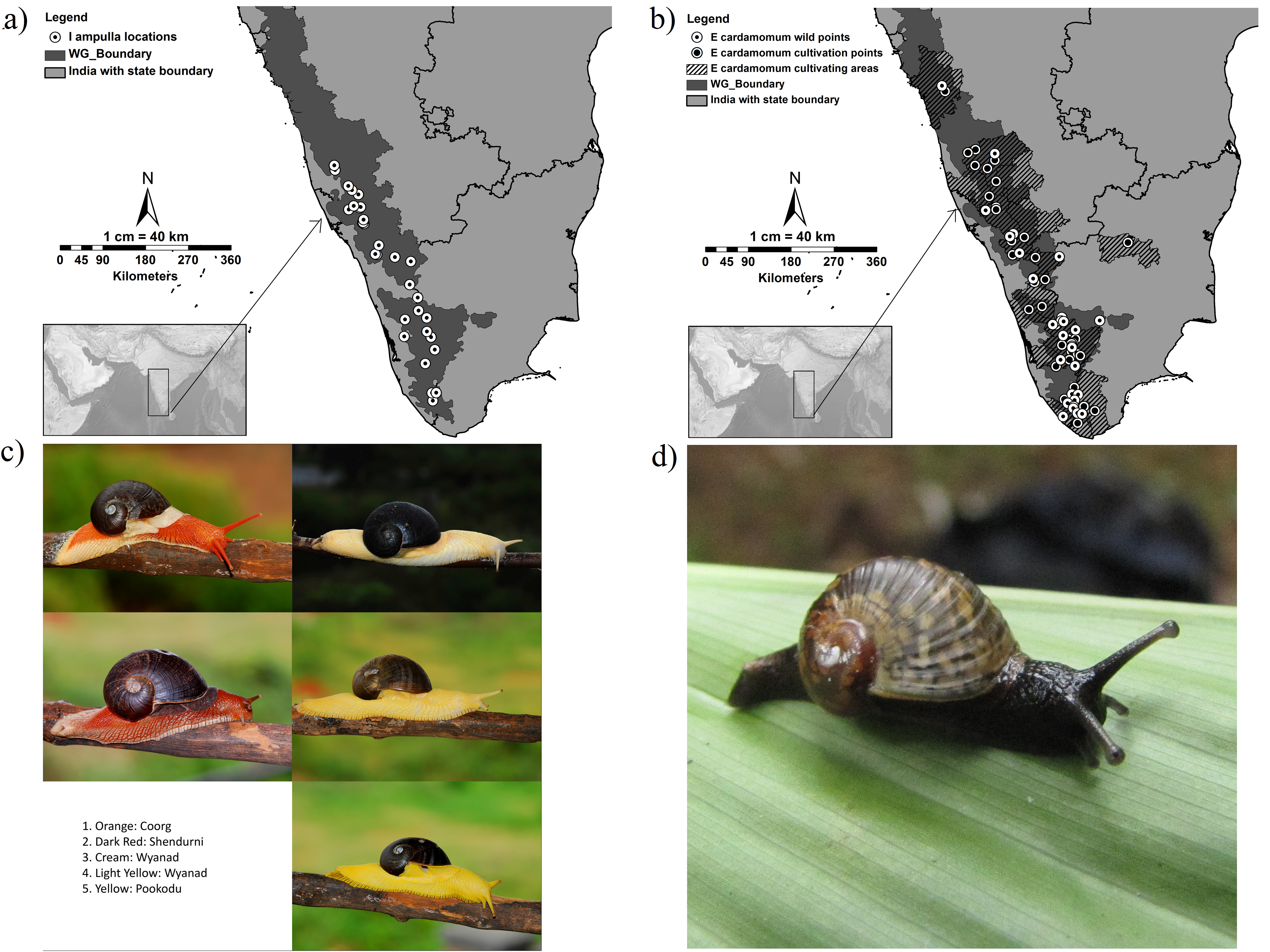


**Table S2:** The different treatment and number of occurrence records used to calibrate the *E. cardamomum* model.

| **Type of treatment used to calibrate the model** | | **Number of records** | | |
| --- | --- | --- | --- | --- |
| **Bias file type** | **Treatments used** | **Train** | **Test** | **Total** |
| All (wild + cultivated) | All records | 47 | 20 | 67 |
|  | Cultivated records only | 26 | 11 | 37 |
|  | All cultivated records + 50% of wild records | 37 | 15 | 52 |
|  | Wild records only | 20 | 10 | 30 |
|  | All wild records + 50% of cultivated records | 34 | 14 | 48 |
| Cultivated only | All records | 47 | 20 | 67 |
|  | Cultivated records only | 26 | 11 | 37 |
|  | All cultivated records + 50% of wild records | 37 | 15 | 52 |
|  | Wild records only | 20 | 10 | 30 |
|  | All wild records + 50% of cultivated records | 34 | 14 | 48 |
| Wild only | All records | 47 | 20 | 67 |
|  | Cultivated records only | 26 | 11 | 37 |
|  | All cultivated records + 50% of wild records | 37 | 15 | 52 |
|  | Wild records only | 20 | 10 | 30 |
|  | All wild records + 50% of cultivated records | 34 | 14 | 48 |

**Appendix S2. Detailed Methodology of creating bias file for *Indrella ampulla* and *E cardamomum***

Most of the time, the sampling of a species across its geographic distribution is not random or systematic sampling efforts are always geographically biased towards more easily accessed or better-surveyed areas (Phillips *et al*. 2009; Ruiz-Gutierrez & Zipkin 2011). Furthermore, the increased use of specimen locality data available from open access data portals in combination with other data sources exacerbates over-representation of some regions within a study area, which can cause a severe spatial bias in the collected occurrence data (Hortal *et al*. 2008). As a result, the sampling locations of a species may not be representative of the true range of environmental conditions in which the species occurs (Reddy & Davalos 2003) and leads to environmental bias because of the over-representation of certain environmental features of the more accessible and extensively surveyed areas. Thus, failure to correct for geographical sampling bias can result in a habitat suitability models that reflects sampling effort rather than the true distribution of a species (Ruiz-Gutierrez & Zipkin 2011). Therefore, it is very important to account for spatial sampling bias to predict true distribution of habitat for a species (Elith *et al*. 2010; Kramer-Schadt *et al*. 2013).

In the present study, some parts of Western Ghats have been more intensively sampled for *I. ampulla* and *E. cardamomum* than others. To control for this sample selection bias (Ruiz-Gutierrez & Zipkin 2011), we assigned greater weight to *I. ampulla* and *E. cardamomum* presence-only points with fewer neighbors in geographic space by creating a bias grid (Elith *et al.* 2010). As the study does not have temporal records of sampling effort from studies that compiled the presence only data, we cannot distinguish between areas that are environmentally unsuitable and those that are under sampled. The detailed methodology used to create bias grids for the species as follows.

1. *Raster and point conversion of presence records*: we converted one set of presence records of *I. ampulla* and three sets of presence records (wild records only, cultivated records only and all records) of *E. cardamomum* to raster using ArcMap. We used one of the environmental layer prepared for MaxEnt model as a reference layer in environmental settings options to have same geographic projection and cell size for raster. Then we converted this raster back to points to remove the duplicate points and to obtain cell center.
2. *Creation of background points*: to create background points, we first generated minimum convex polygons separately for *I. ampulla* and *E. cardamomum* using the points created in step 1. We used *Minimum Bounding Geometry* option in *Data Management Tools* extension of ArcMap, ArcGIS 10.0 to create minimum convex polygons. We converted these polygons to raster using same reference layer as that of step 1 to have same geographic projection and cell size for raster. Then we converted raster to points and added XY coordinates using *Add XY Coordinates* option in *Data Management Tools* extension of ArcMap, ArcGIS 10.0. Finally, we generated 10000 random background points from XY coordinates added points using *Create Random Points* option in Data Management Tools extension of ArcMap, ArcGIS 10.0. In total we generated 4 different layers of background points, one for *I. ampulla* and three for *E. cardamomum* (because *E cardamomum* had three different sets of occurrence records).
3. *Combining background points with presence records*: we combined 10000 background points created separately for each species in step 2 with presence points created for each species in step 1.
4. *Converting combined background points and presence records to equidistant projection*: we converted combined background points created in step 3 and presence points created in step 1 to *Asian South Albers Equal Area Conic* using *Projections and Transformations* option in Data *Management Tools* extension of ArcMap, ArcGIS 10.0. Finally, we converted projected points from meters to kilometers using Calculate Geometry option in ArcMap, ArcGIS 10.0.
5. *Calculating distance between combined background points and presence records:* we calculated distance between equidistant projected combined background points and presence records created in step 4 using *Geospatial Modelling Environment* *(GME)(*[*http://www.spatialecology.com/game/*](http://www.spatialecology.com/game/)*)* tool. This tool gives output in .csv file format.
6. *Calculation of Gaussian kernels:* using ArcCatalog we exported .csv file obtained in step 5 to .dbf file and opened converted .dbf file in ArcMap and added new field using option *Add Field*. In the new field using *Field Calculator* option, we applied *Gaussian* function:

exp ( - ([d]^2)/(2*s^2)),

Where d = distance calculated in kilometers in step 5 and s = standard deviation of species depending on species home range and or sampling methods (23). We chose a standard deviation of 3.5 km for *I. ampulla* because during our field survey, we found *Indrella* populations frequently within 12 to 13 kilometers area in a particular location. Therefore, a circular area has a diameter of approximately 3.5 km. In case of *E. cardamomum* we used standard deviation of 0.5 km because during our field survey, we encountered plantations after every 1.5 to 2 kilometers in a particular location. Once, Gaussian function calculated we summed Gaussian function by right clicking on new field and choosing *Summarize*. Note: To use bias grid in MaxEnt, we want to upweight sites in sparse areas, so used a reciprocal of the summed Gaussian values scaled to vary between 1 and 25.

1. *Joining summed Gaussian kernels with combined background points: To develop the shape file for the bias grid,* we joined summed Gaussian kernels with combined background points created in step 3 using *Join* function in ArcMap and joined based on FID and OID columns of two layers. Then we exported joined layer to shape file by right clicking on *Table of Contents* and choosing *Data Export* option in ArcMap~~.~~
2. *Converting shapefile of bias grid to .asc format*: we converted shapefile of bias grid to .asc format using ArcMap, ArcGIS 10.0. Before converting shapefile to .asc format, we projected shapefile to geographic projection WGS1984 and used same environmental layers as reference layer as that of step 1 to have same geographic projection and cell size for raster. Finally, we had four bias grid files one for *I ampulla* and three for *E cardamomum* (Fig. S2a to S2d).

**Figure S2.** Bias grids created for *I. ampulla* and *E. cardamomum* a) bias grid of *I. ampulla* b) bias grid of *E. cardamomum* created using all (both cultivated and wild) records c) bias grid of *E. cardamomum* created using only cultivated records d) bias grid of *E. cardamomum* created using only wild records (see Appendix S1 for how these bias grids were created).


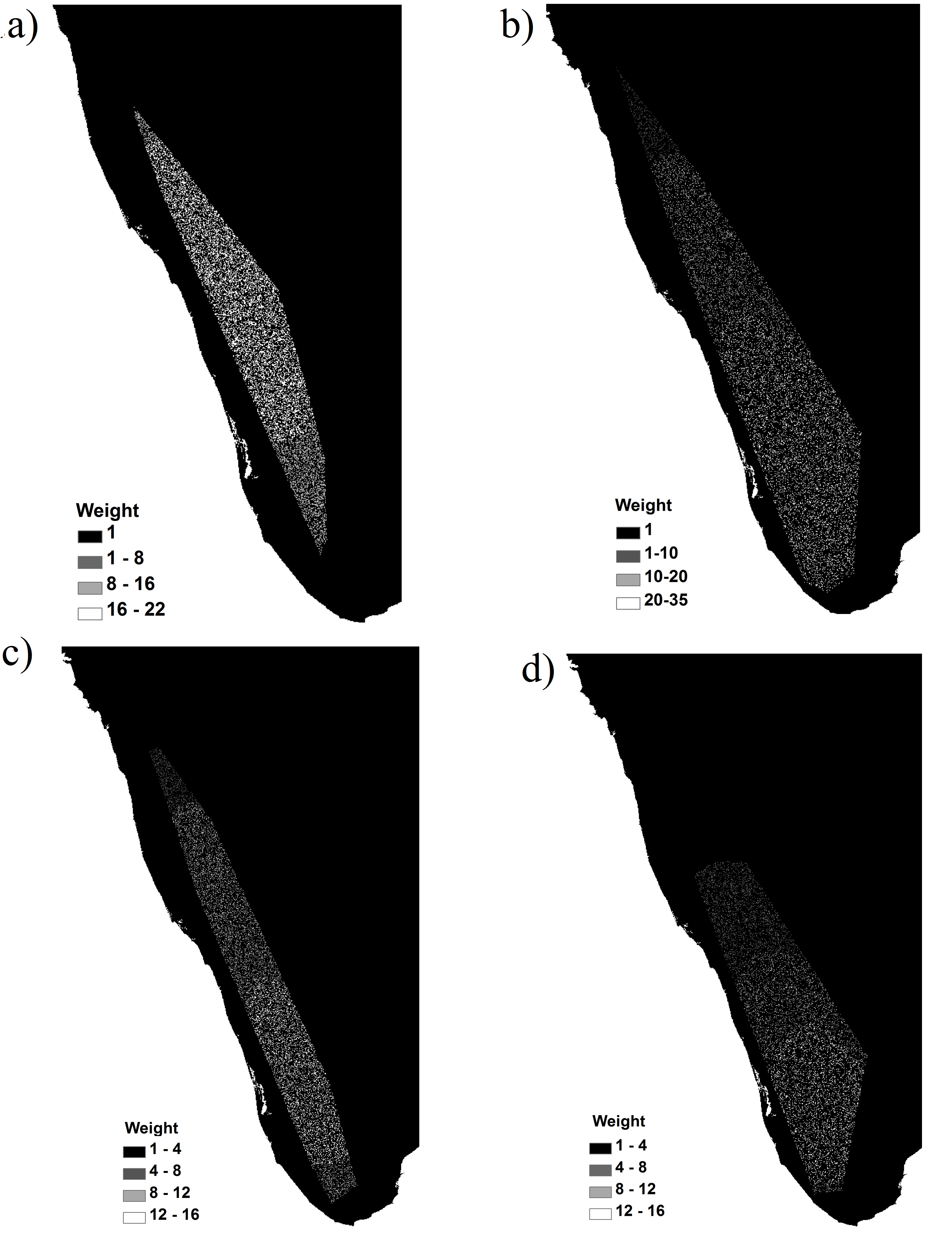


**Appendix S3: Results and discussion of Important predictors of habitat suitability for *I ampulla* and *E. cardamomum*.**

In case of *E. cardamomum*, among 21 environmental variables 5 variables together contributed to 91.13% variation for habitat suitability model of *E cardamomum*. Isothermality (46.90%) and Precipitation of warmest quarter (17.41%) had highest contribution followed by Maximum temperature of warmest month (15.22%), Global aridity index (6.53%) and Elevation (5.03%) (Table 1). Considering permutation importance, the same variables except elevation had maximum influence on habitat suitability model of *E. cardamomum* (Table 1). The future distribution model of *E. cardamomum* was also influenced by same variables, except global aridity index that was replaced by Precipitation seasonality and together contributed 83.93 % of variation to the model (Table 1). It was also seen that, the high probability of predicting suitable areas for *E*. *cardamomum* distribution in Western Ghats were associated with these five variables as indicated by response curves (Fig S3a to S3e). The high values of training and test gain and high-test AUC values for the variables further confirmed that these variables had important influence on distribution model of *E. cardamomum* (Fig S3f to S3h).

However, there may be other abiotic and biotic variable, which influence distribution and invasion of *I. ampulla* to *E. cardamomum* plantations in Western Ghats. *I. ampulla* prefers to live in bioclimatically stable habitat like rain forests of Western Ghats, where there will not be many fluctuations in climatic factors such as rainfall, temperature etc. Nevertheless, due to growing demands of human population, most of its habitat has been taken over by coffee and cardamom planters. Interestingly, most of the places where *Indrella* is distributed are also very suitable for growing cardamom. However, our results also confirm that, the same environmental variables, which are predicted to be important for *I. ampulla* distribution, were also major predictors of potential suitable areas of *E. cardamomum* cultivation in Western Ghats (Table 1, Fig 3a to 3e and Fig S4a to S4e). As both the species require same environmental conditions, it is possible that why we frequently encountered most of the populations of *I. ampulla* in cardamom plantations near to forest and only few populations in forest during our survey. Our study also suggested that, the population density positively influenced by canopy cover and litter depth (Table S3), but day-by-day reduction of canopy cover in natural forest due to their conversion into cardamom plantations, many of the *I.ampulla* populations were observed to resting under the canopy of cardamom plants. Snails feed on leaf litter and fungi but due to lack of litter under cardamom plants and limited growth of fungi in cardamom plantations due to extensive use of herbicides and fungicides, there may not be enough supply of food to *Indrella* in cardamomum plantations and hence, it might have specialized to feed on cardamom flowers and berries. One more reason why the *Indrella* might have invaded cardamom plantations is, supply of calcium, planters use tons of calcium carbonate to control blight diseases of fungi and to reduce soil acidity. Calcium is very important for bodily process; reproduction and most notably shell production of snail (Fournié & Chetail 1984; Burch & Pearce 1990). Due to this reason, they might have invaded the cardamom plantations, where calcium supply is more and easily available compared to natural forest where snails have to obtain calcium by ingesting calcium particle from soil particles, rasping calcium rich rocks and digesting decaying leaf matter (Fournié & Chetail 1984; Burch & Pearce 1990; Wäreborn 1979, 1992). Our results also confirmed that cation exchange capacity (CEC) and calcium carbonate (CaCO3) content of top soil is very important for *I. ampulla* distribution (Table 1).

**Table S3:** Relationship of snail density with microhabitat parameters and extant of disturbance noticed. * indicates p-value significant at 0.05.

| **Habitat quality parameters** | **r-value** | **p-value (2-tailed)** |
| --- | --- | --- |
| Height at which it is found | 0.297 | 0.036* |
| Litter depth in cm | 0.260 | 0.068 |
| Canopy cover | 0.340 | 0.016* |
| Litter cover | 0.116 | 0.422 |
| No of trees inside the quadrat | 0.084 | 0.560 |
| No of dead logs found | 0.014 | 0.925 |

**Figure S3.** Marginal response curves of the predicted probability of suitable habitat of *E. cardamomum* in Western Ghats for five environmental variables that contributed substantially to the MaxEnt model and jackknife analysis of individual predictor environmental variables important in the development of full habitat suitability model for *E. cardamomum* a) precipitation of coldest quarter b) isothermality c) maximum temperature warmest month d) elevation e) Aridity index f)training gain for environmental variables g) test gain for environmental variables and h) test AUC for environmental variables.


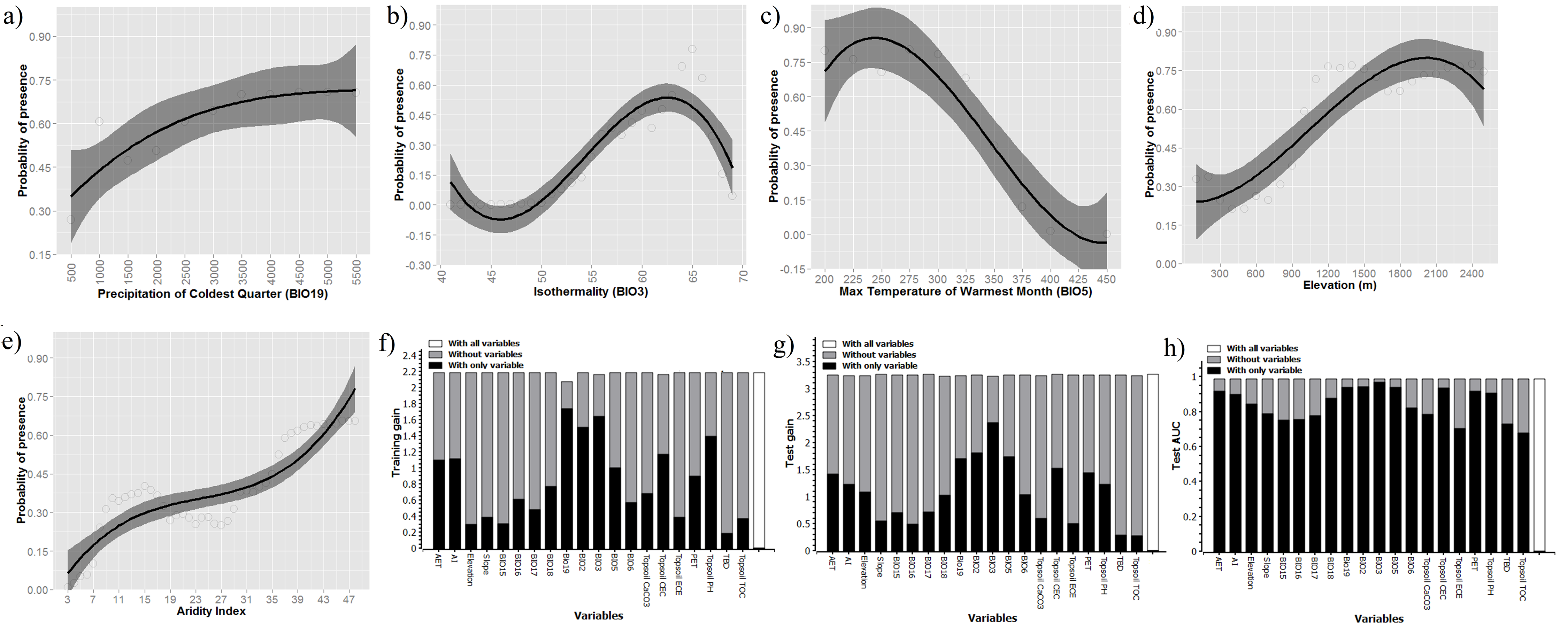


Note: Dark grey area on either side of response curve indicates the 5 to 95% confidence intervals from 50 replicated models. See Table S1 for percent contributions of each predictor environmental variables (i.e. variable importance) to MaxEnt habitat suitability model of *E cardamomum* in Western Ghats. In the jackknife analysis, black bars indicate the training gain, test gain and AUC achieved in the jackknife results of models when including only that variable and excluding the remaining variables; grey bars show how much the training gain, test gain and AUC is diminished without the given predictor variable and white bar indicates training gain, test gain and AUC achieved with all the variables included in model.

**Figure S4.** Marginal response curves of the predicted probability of suitable habitat of *I. ampulla* in Western Ghats for five environmental variables that contributed substantially to the MaxEnt model and jackknife analysis of individual predictor environmental variables important in the development of full habitat suitability model for *I. ampulla* a) precipitation of coldest quarter b) isothermality c) topsoil cation exchange capacity d) elevation e) Aridity index f) training gain for environmental variables g) test gain for environmental variables and h) test AUC for environmental variables.


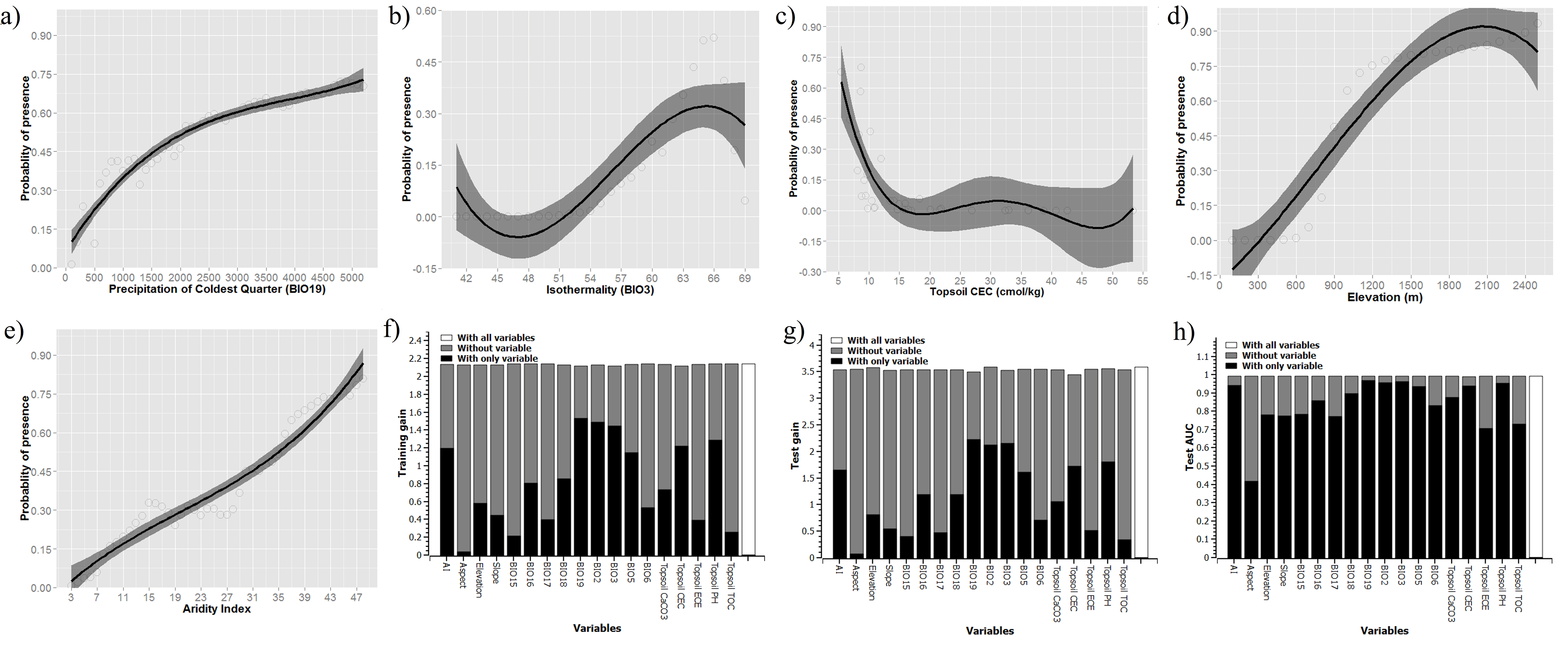


Note: Dark grey area on either side of response curve indicates the 5 to 95% confidence intervals from 50 replicated models. See Table 1 for percent contributions of each predictor environmental variables (i.e. variable importance) to MaxEnt habitat suitability model of *E. cardamomum* in Western Ghats. In the jackknife analysis, black bars indicate the training gain, test gain and AUC achieved in the jackknife results of models when including only that variable and excluding the remaining variables; grey bars show how much the training gain, test gain and AUC is diminished without the given predictor variable and white bar indicates training gain, test gain and AUC achieved with all the variables included in model.

**Appendix S4: Result and discussion for Model calibration of *E. cardamomum***

Model calibration Result of *E. cardamomum*: In case of *E. cardamomum*, first we calibrated model using 15 different treatments, which included different set of wild and cultivated records and three different bias files (Table S2 and Fig S2b to S2d). The average AUC values were high for all the treatments and not significantly differed across treatments and we were unable to choose singe best performing model of *E. cardamomum* (Table S4). Then we decided to choose the best-performing model of *E. cardamomum* among 15 different treatments by calculating average pair-wise similarity among models and chosen the models developed using all cultivated records plus 50% of randomly chosen wild records with bias file created using only cultivated records (Table S2) to perform further analysis. Because this model had highest average similarity with all other models and with average consensus model derived from all 15 treatment (Table S4). The finally chosen model of *E. cardmomum* performed best in both climatic scenario (current and future (2080)), because they had high average values of AUC and TSS obtained from 50 replicated models (evaluated using 30% of records) ((current: AUCTRAIN = 0.991±0.000, AUCTEST = 0.986±0.013; future: AUCTRAIN = 0.991±0.000, AUCTEST = 0.986±0.014) and (TSS: current = 0.962±0.010; future = 0.961±0.012)) (Table 1).

Our results also highlight the importance of accounting for sampling bias when modelling potentially suitable areas for cultivation of native crops. There were only few cultivated records (n=37) of *E. cardamomum* in Western Ghats. However, cardamom is cultivated in more than 37 locations in Western Ghats. Moreover, the maximum number of cultivated and wild records was obtained from southern Western Ghats, and only few records were from central and northern part of Western Ghats (Fig S1b). For this reason, we believe that the cultivated range of *E. cardamomum* is not sufficiently captured and there was an oversampling of records from southern Western Ghats (Fig S1b). To account for sampling bias and to effectively capture the suitable areas of *E. cardamomum* cultivation in Western Ghats, we used bias file as recommended (Elith *et al*. 2010) and the model prediction was better after accounting for sampling bias.

Discussion on model calibration of *E. cardamomum*: In case of *E. cardamomum*, the model was calibrated using both wild and cultivated range records and accounting for sampling bias. Mau-Crimmins *et al.* 2006 and Beaumont et al. 2009 evaluated the importance of calibrating models using either native range data (wild) or invaded range data. Models tend to perform better when calibrated using invaded range data than native (wild) range data (Crimmins *et al*. 2006; Beaumont et al. 2009). Models calibrated using invaded range data can consider factors not present in the native range, such as environmental preferences of the invaded range genotype (Beaumont *et al*. 2009). The results of *E. cardamomum* model calibration using wild and cultivated range data contradicts the results of Beaumont et al. 2009, we did not find significant difference in model performance among models calibrated using either wild range data or cultivated range data or not even accounting for sampling bias towards cultivated and wild range data (for all the treatments AUC was > 0.9). It is probably because the *E. cardamomum* is native to Western Ghats and most of the cardamom cultivation areas located in its native range Western Ghats, as a result the environmental preferences for both wild and cultivated *E. cardamomum* genotypes may not differ much. However, the model calibrated using all cultivated records and 50% of wild records with accounting sampling bias for cultivated records produced best model of habitat suitability for *E. cardamomum* cultivation in Western Ghats than other treatments. It appears that including both cultivated and wild records can effectively model the suitable areas for cultivation of native crops domesticated in its native range than alone using either wild or cultivated records.

**Table S4:** The area under curve (AUC) values and average similarity of different treatments used to calibrate the *E.cardamomum* model.

| **Type of treatment used to calibrate the model** | | **AUC value** | | **Average similarity of calibrated model** | |
| --- | --- | --- | --- | --- | --- |
| **Bias file type** | **Treatments used** | **Train** | **Test** | **To other models** | **To average of all models** |
| All (wild + cultivated) | All records | 0.988(0.000) | 0.986(0.009) | 0.913 | 0.974 |
|  | Cultivated records only | 0.990(0.000) | 0.985(0.011) | 0.903 | 0.964 |
|  | All cultivated records + 50% of wild records | 0.990(0.000) | 0.987(0.011) | 0.907 | 0.968 |
|  | Wild records only | 0.995(0.000) | 0.993(0.006) | 0.865 | 0.919 |
|  | All wild records + 50% of cultivated records | 0.992(0.000) | 0.988(0.010) | 0.916 | 0.974 |
| Cultivated only | All records | 0.991(0.000) | 0.987(0.012) | 0.930 | 0.980 |
|  | Cultivated records only | 0.992(0.000) | 0.987(0.011) | 0.918 | 0.976 |
|  | All cultivated records + 50% of wild records | 0.991(0.000) | 0.986(0.013) | **0.931** | **0.985** |
|  | Wild records only | 0.995(0.000) | 0.992(0.009) | 0.782 | 0.821 |
|  | All wild records + 50% of cultivated records | 0.993(0.000) | 0.990(0.009) | 0.895 | 0.940 |
| Wild only | All records | 0.991(0.000) | 0.988(0.009) | 0.912 | 0.969 |
|  | Cultivated records only | 0.995(0.000) | 0.989(0.015) | 0.857 | 0.907 |
|  | All cultivated records + 50% of wild records | 0.993(0.000) | 0.988(0.016) | 0.901 | 0.953 |
|  | Wild records only | 0.996(0.000) | 0.993(0.007) | 0.898 | 0.947 |
|  | All wild records + 50% of cultivated records | 0.994(0.000) | 0.990(0.010) | 0.921 | 0.973 |

Note: Values in parenthesis represent standard deviation. Model with highest similarity is indicated in bold.

**Appendix S5: Detailed methodology and discussion of combining predicted habitat suitability and demographic parameters of *I. ampulla***

Methodology: The potential invasion of any pest or invasive species depends on several factors, including quality of the habitat invaded and establishment success of invader, which is measured by demographic parameters of population such as reproduction rate (number of propagules or juveniles produced/adult) and density of adults (they represent number of potential breeding individuals present in population) (Matlaga & Davis 2013, Aguliar *et al*. 2014). Recently many studies have reported that the quality of the habitat significantly influence establishment success and fitness of population (Yañez *et al*.2012; Nagaraju *et al*. 2013). It is predicted that, the species populations located in optimal (highly suitable) habitat should have higher establishment success by producing more propagules and hosting higher number of adults compare to marginal (poor or unsuitable) habitat thus contributing to higher fitness to the population located in highly suitable habitat (Yañez *et al*.2012; Nagaraju *et al*. 2013). Here in case of *I. ampulla*, we predict that *I. ampulla* populations located in highly suitable habitat should have higher number of adults and highest reproduction rate (juveniles/adult) compare to populations located in poor or unsuitable habitat. Thus, we expect any cardamom cultivation area, which overlaps with highly suitable habitat of *I. ampulla* in Western Ghats, should be more vulnerable to higher establish success and invasion of *I. ampulla*. To evaluate the above prediction, we combine predictions of habitat suitability models with demographic parameters of *I. ampulla*. First, we extracted habitat suitability values (ranges from 0 to 1) for each of the eight-selected known population of *I. ampulla* in Western Ghats and converted these continuous probability values of habitat suitability to three categorical classification (highly suitable, medium and poor) using the threshold maximum sensitivity plus specificity (Table S5). Then, each of the eight populations were assigned to these three categories (highly suitable, medium and poor or unsuitable) (Table S5). Secondly, at each of the above eight sites, 3 to 15 quadrats (10*10) were laid and data on demographic parameters such as density of adults per quadrat (which included both live snails and shells of recently dead snails) and the number of juveniles/quadrat were collected. As a measure of reproduction rate, we computed the ratio of number of juveniles to the total number of adult snails per quadrat. Finally, the differences in the various demographic parameters across different habitat suitability categories (highly suitable, medium and poor) were analyzed using Student’s t-test and compared with the prediction.

Discussion: Our results indicated that, the cardamom plantations that overlapped with highly suitable habitat of *I. ampulla* had high density of adults and juveniles of *I. ampulla* and thus higher reproductive potential. For example, the cardamom plantation from Rajakkad located in Kerala state (southern Western Ghats), had highest density of adults and juveniles of *I. ampulla* and also had higher reproductive potential and our model predicted that this particular location overlaps with one of the highly suitable habitat of *I. ampulla* and had highest habitat suitability values (Table S5). Moreover, the *I. ampulla* individuals collected from this region had highest egg laying capacity (the two individuals collected and monitored in captivity laid 130 eggs together) compare to individuals collected from Koppati, Coorg (two individuals together laid 35 eggs). Indicating *I. ampulla* populations located in Rajakkad had highest reproductive fecundity and have potential to become a pest in this region. Interestingly, from the same location recently *I. ampulla* has been reported as pest (Sudhi 2010).

**Table S5:** The eight *I. ampulla* populations chosen for collecting demographic data with their respective habitat suitability, No of quadrats surveyed and demographic parameters

| **Population name** | **Habitat suitability value** | **Habitat suitability category** | **Demographic parameters (Mean±SE)** | | | **No of quadrats surveyed** |
| --- | --- | --- | --- | --- | --- | --- |
|  |  |  | **Adults/quadrat** | **Juveniles/quadrat** | **Juveniles/adult** |  |
| Rajakkad | 0.823 | Highly suitable | 6.400(0.877) | 9.467(2.305) | 1.618(0.510) | 15 |
| Koppati | 0.791 | Highly suitable | 1.500(0.267) | 0.375(0.263) | 0.250(0.164) | 8 |
| Pookodu | 0.799 | Highly suitable | 1.000 (0.577) | 0.333(0.333) | 0.333(0.333) | 3 |
| Schendurney | 0.682 | Medium | 0.353(0.125 | 0.000(0.000) | 0.000(0.000) | 8 |
| Katlapara swamp | 0.691 | Medium | 0.600(0.400) | 0.000(0.000) | 0.000(0.000) | 5 |
| Poonchola | 0.695 | Medium | 2.000(1.154) | 0.667(0.667) | 0.167(0.167) | 3 |
| Vythri | 0.516 | Poor | 1.300(0.335) | 0.400(0.221) | 0.350(0.211) | 10 |
| Abbey falls | 0.583 | Poor | 0.000(0.000) | 0.000(0.000) | 0.000(0.000) | 3 |

**Appendix S6: Detailed methodology for predicted habitat overlap for *I. ampulla* and *E. cardamomum* in future climate scenario.**

*Method:* We also developed maps of cardamom cultivation areas predicted to overlap with highly suitable habitat of *I. ampulla* in future climate (2020 to 2080) scenario. To do this we converted habitat suitability maps of *I. ampulla* to presence-absence suitability map using the maximum sensitivity plus specificity threshold (Table 1). Then we superimposed presence-absence suitability maps of *I. ampulla* with best-performed presence-absence suitability maps of *E. cardamomum* ( future (2020 to 2080)) maps of habitat suitability) to predict the cardamom cultivation areas overlapped with suitable habitat of *I. ampulla* and to determine sensitivity of cardamom cultivation areas to *I. ampulla* invasion in future climatic scenario.

**Figure S5**. Distribution of available suitable habitat for *I. ampulla* in Western Ghats as modelled by Maxent under the assumption of future climate (2020 to 2080) scenario: The maps of habitat suitability for future climate scenario represent the consensus of two general circulation models (CSIRO_MK3 and UKMO_HADCM3) for two SRES emission scenario (B1 and A2) a) 2020 A2 b) 2040 A2 c) 2060 A2 d) 2080 A2 e) 2020 B1 f) 2040 B1 g) 2060 B1 h) 2080 B1

Note: The logistic outputs of consensus Maxent models were transformed to three categorical classifications (highly suitable, medium and poor or unsuitable using the Maximum Training Sensitivity plus Specificity (MTSS) threshold. The dark red shades indicate highly suitable habitat and light cream shades indicate poor or unsuitable habitat for *I. ampulla* distribution in Western Ghats.

**
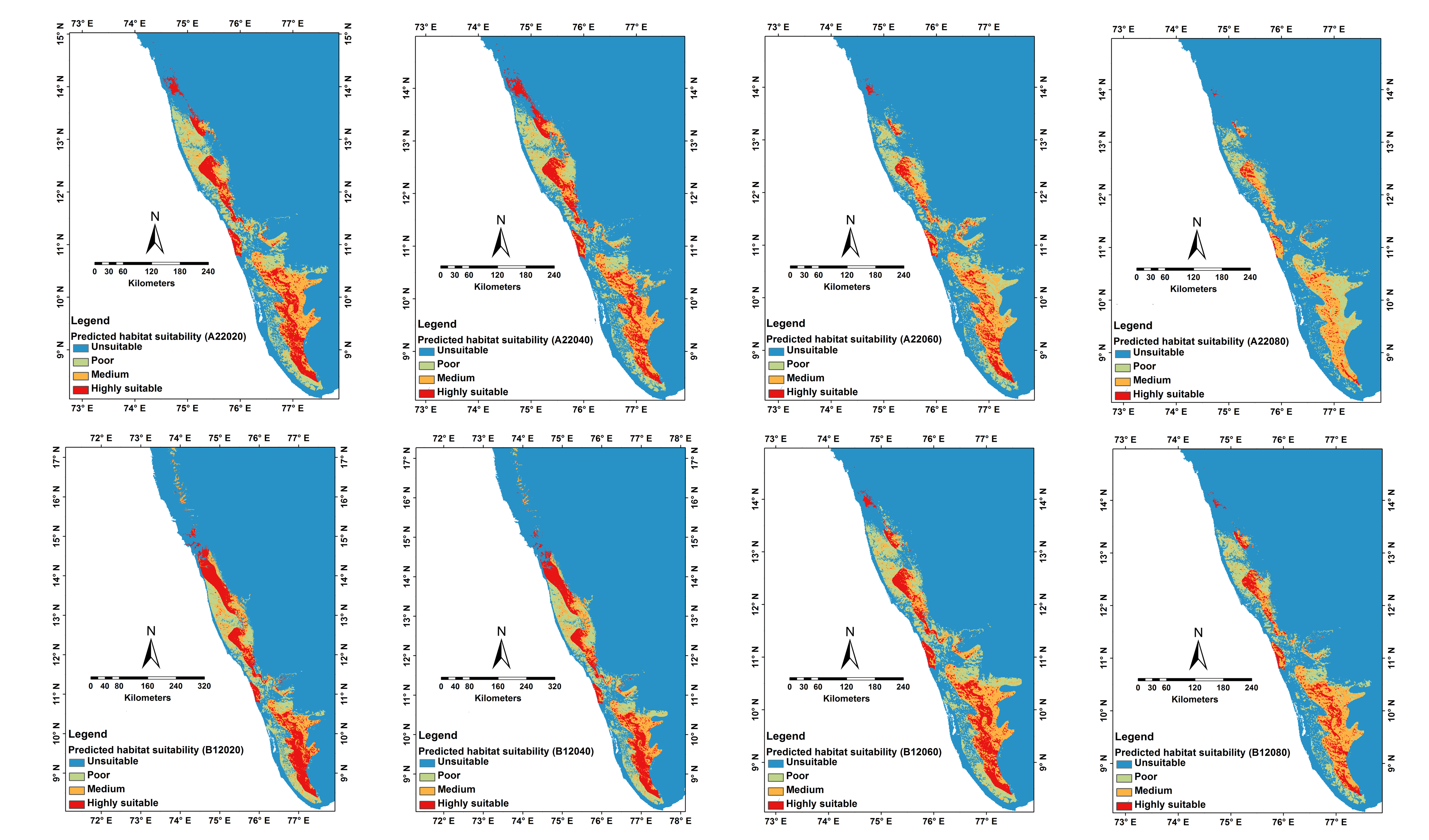
**

**Figure S6.** Distribution of available suitable habitat for *E. cardamomum* cultivation in Western Ghats as modelled by Maxent under the assumption of future climate (2020 to 2080) scenario. The maps of habitat suitability for future climate scenario represent the consensus of two general circulation models (CSIRO_MK3 and UKMO_HADCM3) for two SRES emission scenario (B1 and A2) a) 2020 A2 b) 2040 A2 c) 2060 A2 d) 2080 A2 e) 2020 B1 f) 2040 B1 g) 2060 B1 h) 2080 B1

Note: The logistic outputs of consensus Maxent models of future climate scenario were transformed to three categorical classifications (highly suitable, medium and poor or unsuitable using the Maximum Training Sensitivity plus Specificity (MTSS) threshold. The dark red shades indicate highly suitable habitat and light cream shades indicate poor or unsuitable habitat for *E. cardamomum* cultivation in Western Ghats.

**
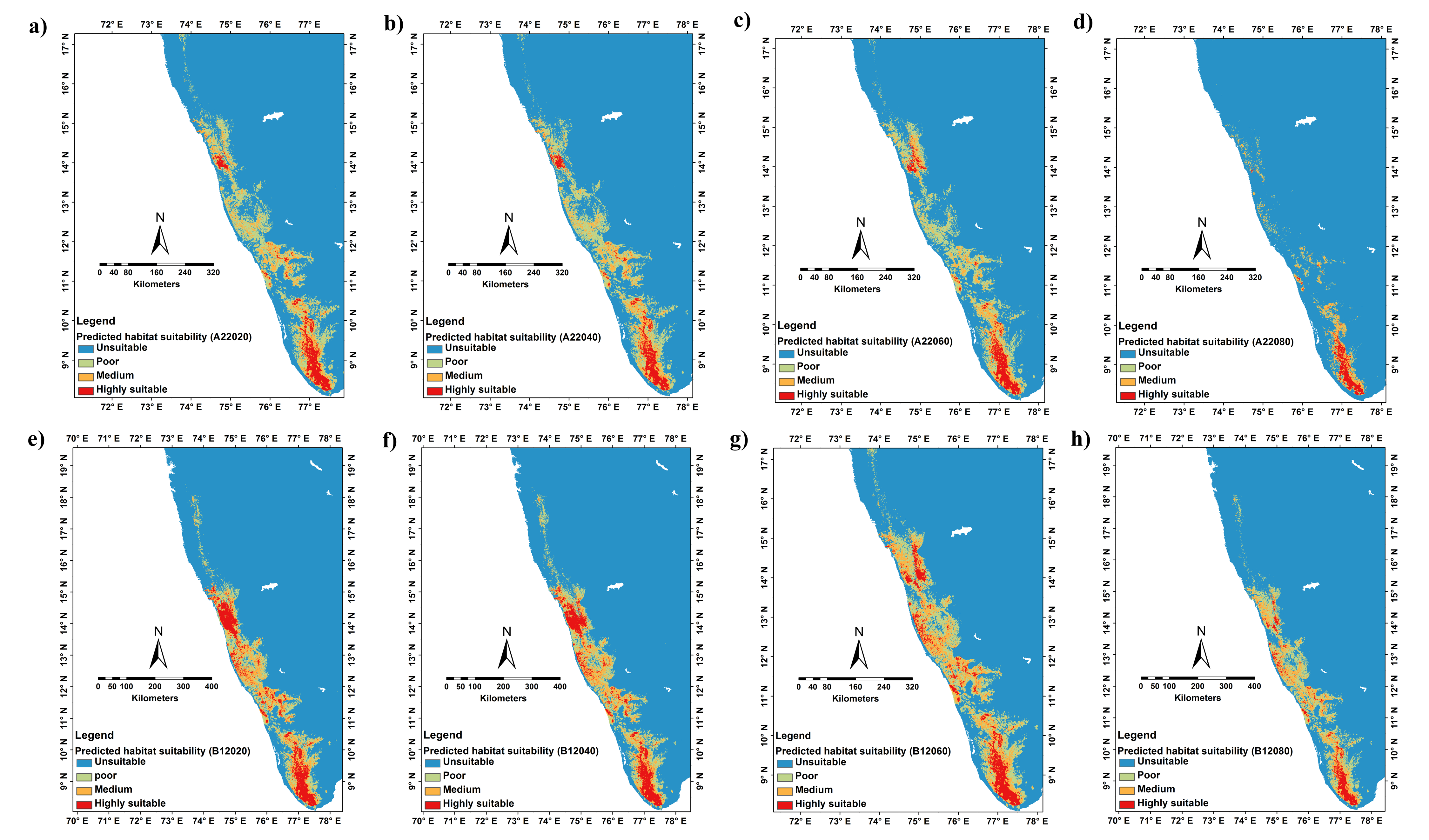
**

**Figure S7.** The map of potential habitat overlap of *E. cardamomum* cultivation areas and *I. ampulla* in Western Ghats as modelled by Maxent under the assumption of future climate (2020 to 2080) scenario. The maps for future climate scenario represent the consensus of two general circulation models (CSIRO_MK3 and UKMO_HADCM3) for two SRES emission scenario (B1 and A2) a) 2020 A2 b) 2040 A2 c) 2060 A2 d) 2080 A2 e) 2020 B1 f) 2040 B1 g) 2060 B1 h) 2080 B1.

Note: The logistic outputs of consensus Maxent models of future climate scenario were transformed to binary (presence-absence) classifications using the Maximum Training Sensitivity plus Specificity (MTSS) threshold.


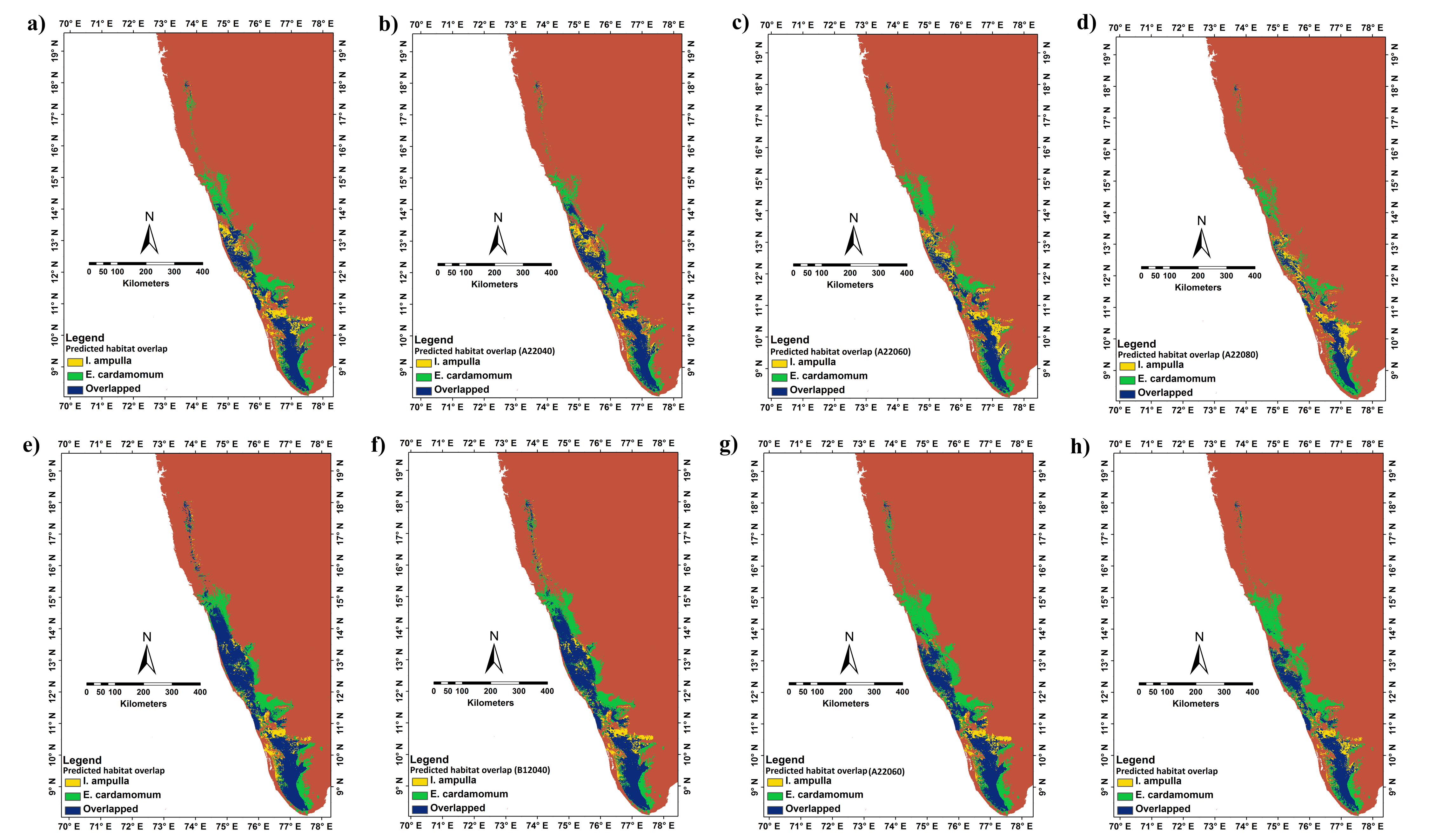


**References**

Allouche, O., Tsoar, A & Kadmon R. (2006) Assessing the accuracy of species distribution models: prevalence, kappa and the true skill statistic (TSS). *Journal of Applied Ecology* **43**: 1223–1232.

Araújo, M.B & New, M. (2006) Ensemble forecasting of species distribution. *Trends in Ecology and Evolution* **22**: 42–47.

Beaumont, L.J., Gallagher, R.V., Thuiller, W., Downey, P.O, et al. (2009) Developing climatic envelopes among invasive populations may lead to underestimations of current and future biological invasions. *Diversity and Distributions* **15**: 409–420.

Burch, J.B & Pearce, T.A. (1990) *Terrestrial gastropoda*. *Soil Biology Guide* (eds. D.L. Dindal), pp. 201-299, John Wiley & Sons, Inc., New York, NY.

Christensen, J.H., Hewitson, B., Busuioc, A., Chen, A., Gao, X., Held, I., Jones, R., Kolli, R.K., Kwon, W.T., Laprise, R., Magaña Rueda, V., Mearns, L., Menéndez, C.G., Räisänen, J., Rinke, A., Sarr, A & Whetton, P. (2007) *Regional Climate Projections. In: Climate Change 2007: The Physical Science Basis. Contribution of Working Group I to the Fourth Assessment Report of the Intergovernmental Panel on Climate Change* (eds S.D. Solomon., M. Qin., Z. Manning., M. Chen., M. Marquis., K.B. Averyt., M. Tignor & H.L. Miller), Cambridge University Press, Cambridge, United Kingdom and New York, NY, USA.

Cuest-Comocho, F., Ganzenmuller, A., Peralvo, M., Novoa, J. & Riofrio, G. (2006) *Predicting species’ niche distribution shifts and biodiversity change within climate change scenarios: A regional assessment for bird and plant species in the Northern Tropical Andes. Peru:* Biodiversity Monitoring Program, EcoCiencia.

Elith, J., Kearney, M. & Phillips, S. (2010) The art of modelling range-shifting species. *Methods in Ecology and Evolution,* **1**, 330–342.

ESRI (2012) ArcGIS Desktop Release 10. Redlands, CA, Environmental Systems Research Institute.

Fournié, J & Chetail, M. (1984) Calcium dynamics in land gastropods. *American Zoologist* **24**: 857–870.

Gordon, C., Cooper, C., Senior, C.A., Banks, H., et al. (2000) The simulation of SST, sea ice extents and ocean heat transports in a version of the Hadley Centre coupled model without flux adjustments. *Climate Dynamics* **16**:147–168.

Hortal, J., Jimenez-Valverde, A., Gomez, J.F., Lobo, J.M. & Baselga, A. (2008) Historical bias in biodiversity inventories affects the observed environmental niche of the species. *Oikos*, **117**, 847–858.

IPCC. (2000) Special Report on Emissions Scenarios (SRES). Working Group III Intergovernmental Panel on Climate Change 21.

Jiménez-Valverde, A., Peterson, A., Soberón, J., Overton, J et al. (2011) Use of niche models in invasive species risk assessments. *Biological Invasions* **13**: 2785–2797.

Kadmon, R., Farber, O. & Danin A. (2004) Effect of roadside bias on the accuracy of predictive maps produced by bioclimatic models. *Ecological Applications* **14**: 401–413.

Kramer-Schadt, S., Niedballa, J., Pilgrim, J.D., Schröder, B et al. (2013) The importance of correcting for sampling bias in MaxEnt species distribution models. *Diversity and Distributions* **19**: 1366–1379.

Kumar, BM., Kumar, V.S. & Mathew, T. (1995) Floristic attributes of small cardamom (Elettaria cardamom L. Maton) growing areas in the Western Ghats of Peninsular India. *Agroforestry Systems*, **31**, 275–289.

Kuriakose, G., Sinu, P.A. & Shivanna, K.R. (2009) Domestication of cardamom (*Elettaria cardamomum*) in Western Ghats, India: divergence in productive traits and a shift in major pollinators. *Annals of Botany*, **103**: 727–733.

Liu, C., Berry, P.M., Dawson, T.P & Pearson, R.G. (2005) Selecting thresholds of occurrence in the prediction of species distributions. *Ecography* **28**: 385-393

Liu, C., White, M. & Newell, G. (2009) Measuring the accuracy of species distribution models: a review. *Proceedings 18th World IMACs/MODSIM Congress. Cairns, Australia*, 4241-4247.

Matlaga, D.P. & Davis, A.S. (2013) Minimizing invasive potential of Miscanthus × giganteus grown for bioenergy: identifying demographic thresholds for population growth and spread. *Journal of Applied Ecology* **50**: 479–487.

Mau-Crimmins, T.M., Schussman, H.R. & Geiger, E.L. (2006) Can the invaded range of a species be predicted sufficiently using only native-range data? *Ecological Modelling*, **193**, 736–746.

Nagaraju, S.K., Gudasalamani R, Barve N, Ghazoul J, Narayanagowda GK, et al. (2013) Do ecological niche model Predictions reflect the adaptive landscape of species?: A test using Myristica malabarica Lam., an endemic tree in the Western Ghats, India. *PLoS ONE*, **8(11)**, e82066.

Phillips, S.J., Dudik, M., Elith, J., Graham, C.H., Lehmann, A., Leathwick, J. & Ferrier, S. (2009) Sample selection bias and presence-only distribution models: implications for background and pseudo-absence data. *Ecological Applications*, **19**, 181–197.

Prasath, D. & Venugopal, M.N. (2004) Genetic diversity and conservation of cardamom (Elettaria cardamomum Maton) in India. *Plant Genetic Resource News letter*, **138**: 55–60

Ramirez, J. & Jarvis, A. (2008) High resolution statistically downscaled future climate surfaces. International Center for Tropical Agriculture (CIAT), CGIAR Research Program on Climate Change, Agriculture and Food Security (CCAFS) Cali, Colombia.

Reddy, S. & Davalos, L.M. (2003) Geographical sampling bias and its implications for conservation priorities in Africa. *Journal of Biogeography*, **30**, 1719–1727.

Ruiz-Gutierrez, V. & Zipkin, E.F. (2011) Detection biases yield misleading patterns of species persistence and colonization in fragmented landscapes*. Ecosphere* **2**: 1*–*14*.*

Sanz-Aguilar, A., Anadón, J.D., Carrete, M., Edalaar, P. & Tella, J.L. (2014) Can establishment success be determined through demographic parameters? A case study on five introduced bird species. *PLoS ONE*, **9(10)**, e110019

Stanton, J.C., Pearson, R.G., Horning, N., Ersts, P & Akcakaya, H.R (2011) Combining static and dynamic variables in species distribution models under climate change. *Methods in Ecology and Evolution*, **3**, 349–357.

Sudhi. K.S. (2010) Colorful snails attack cardamom plants, *The Hindu*. Accessed 21 Jan 2015.

Wäreborn, I. (1979) Reproduction of two species of land snails in relation to calcium salts in the foerna layer. *Malacologia* **18**:177–180.

Wäreborn, I. (1992) Changes in the land mollusc fauna and soil chemistry in an inland district in southern Sweden. *Ecography* **15**:62–69.

Yañez-Arenas, C., Martínez-Meyer, E., Mandujano, S. & Rojas-Soto, O. (2012) Modelling geographic patterns of population density of the white-tailed deer in central Mexico by implementing ecological niche theory. *Oikos* **121**: 2081–2089.
